# Survival Outcomes After Multiple vs Single Arterial Grafting Among Patients With Reduced Ejection Fraction

**DOI:** 10.1001/jamanetworkopen.2025.4508

**Published:** 2025-04-10

**Authors:** Justin Ren, Jason E. Bloom, William Chan, Christopher M. Reid, Julian A. Smith, Andrew Taylor, David Kaye, Colin Royse, David H. Tian, Andrea Bowyer, Doa El-Ansary, Alistair Royse

**Affiliations:** 1 Department of Surgery, The University of Melbourne, Melbourne, Australia; 2 Department of Cardiothoracic Surgery, Royal Melbourne Hospital, Melbourne, Australia; 3 Baker Heart and Diabetes Institute, Melbourne, Australia; 4 School of Public Health and Preventive Medicine, Monash University, Melbourne, Australia; 5 Department of Cardiology, Alfred Health, Melbourne, Australia; 6 Department of Cardiology, Western Health, Melbourne, Australia; 7 Department of Medicine, University of Melbourne, Melbourne, Australia; 8 Population Health, Curtin University, Perth, Australia; 9 Department of Surgery, Monash University, Melbourne, Australia; 10 Department of Cardiothoracic Surgery, Monash Health, Melbourne, Australia; 11 Department of Cardiology, Royal Melbourne Hospital, Melbourne, Australia; 12 Department of Medicine, Monash University, Melbourne, Australia; 13 Department of Anesthesia, Royal Melbourne Hospital, Melbourne, Australia; 14 Outcomes Research Consortium, Cleveland Clinic, Cleveland, Ohio; 15 Department of Anesthesia and Perioperative Medicine, Westmead Hospital, Sydney, Australia; 16 School of Biomedical and Health Sciences, RMIT University, Melbourne, Australia; 17 Department of Surgery, Universiti Kebangsaan Malaysia, Kuala Lumpur, Malaysia

## Abstract

**Importance:**

Multiarterial coronary bypass procedures offer improved clinical outcomes compared with single arterial grafting with supplementary saphenous vein grafts. However, the survival advantage of multiarterial grafting across varying levels of left ventricular impairment remains uncertain.

**Objective:**

To compare long-term survival outcomes of patients undergoing multiple vs single arterial grafting, stratified by preoperative ejection fraction.

**Design, Setting, and Participants:**

A complete-case retrospective cohort study was conducted using data from a multicenter population-based cardiac registry established by the Australian & New Zealand Society of Cardiac & Thoracic Surgeons with linkage to the National Death Index. Participants were individuals who underwent primary isolated coronary bypass surgery between June 1, 2001, and January 31, 2020. Exclusion criteria were nonadults, reoperations, concomitant or previous cardiac surgical procedures, single-graft procedure, and cases without any arterial grafts. Statistical analyses were conducted in September 2024.

**Exposures:**

Patients underwent either multiple or single arterial grafting, stratified by their preoperative left ventricular ejection fraction.

**Main Outcomes and Measures:**

Long-term all-cause mortality.

**Results:**

The study included 59 641 patients (mean [SD] age at the time of surgery, 65.8 [10.2] years; 48 321 men [81.0%]). The median follow-up duration was 5.0 years (IQR, 2.3-8.6 years). Multiarterial grafting was associated with a 19.0% relative reduction in all-cause mortality compared with single arterial grafting among patients with a normal left ventricular ejection fraction (hazard ratio [HR], 0.81; 95% CI, 0.75-0.87; *P* < .001). Similar survival benefits were observed among patients with mild (HR, 0.83; 95% CI, 0.77-0.90; *P* < .001), moderate (HR, 0.82; 95% CI, 0.74-0.90; *P* < .001), and severe left ventricular impairment (HR, 0.82; 95% CI, 0.71-0.96; *P* = .01). A multivariable Cox proportional hazards regression interaction-term analysis indicated no significant differences in the multiarterial survival benefit by ejection fraction stratification (*P* = .75). Multiarterial grafting with exclusively arterial conduits was associated with enhanced survival benefits compared with other multiarterial procedures with saphenous vein grafts, except when the left ventricular ejection fraction was below 30% (HR, 0.87; 95% CI, 0.67-1.13; *P* = .30).

**Conclusions and Relevance:**

In this retrospective cohort study using data from a binational database, multiarterial procedures were associated with reduced long-term mortality risk compared with single arterial grafting across the spectrum of preoperative left ventricular ejection fractions. Total arterial revascularization was associated with incrementally improved survival, particularly among patients with preserved ejection fraction. Because most coronary surgery practice continues to use single arterial grafting, consideration to alter grafting strategy to multiarterial procedures may be indicated.

## Key Points

**Question:**

What is the association of left ventricular impairment with survival outcomes of multiple arterial grafting with or without vein vs single arterial grafting?

**Findings:**

In this nationwide cohort study of 59 641 patients with a 5-year median follow-up, multiarterial grafting was significantly associated with improved long-term survival irrespective of preoperative left ventricular ejection fraction. Total arterial revascularization was associated with additional survival benefits compared with other multiarterial procedures.

**Meaning:**

This study suggests that surgeons should prioritize multiple over single arterial grafting, regardless of left ventricular dysfunction, to improve long-term survival, with total arterial revascularization associated with the greatest benefit by eliminating saphenous vein grafts.

## Teaser

This cohort study compares long-term survival outcomes of patients undergoing multiple vs single arterial grafting, stratified by preoperative left ventricular ejection fraction.

## Introduction

Ischemic heart disease remains the leading cause of heart failure with reduced ejection fraction.^1^ Despite the use of state-of-the-art heart failure therapies (both pharmacologic and device based) and the use of contemporary revascularization techniques, the presence of ischemic cardiomyopathy continues to portend a poor prognosis, with 65.0% of patients being deceased within 10 years of the operation.^2^

The use of surgical revascularization with coronary artery bypass grafting (CABG) for patients with ischemic heart failure with reduced ejection fraction is emerging as the preferred mode of revascularization for this patient cohort to improve long-term survival. Conventional CABG practice typically consists of grafting the left internal mammary artery to the left anterior descending coronary artery due to the reported improvement in graft patency, freedom from recurrent cardiac events, and long-term survival.^3,4^ However, in current clinical practice, non–left anterior descending coronary artery targets are almost exclusively bypassed with saphenous vein grafts (SVGs).^5^ This treatment strategy is frequently referred to as *single arterial grafting* (SAG), and may be a problematic grafting strategy given the high rates of venous graft failures, which in prior observational studies have been shown to be associated with increased adverse events.^5^ The use of multiple arterial grafting (MAG)—which will often consist of left internal mammary artery, right internal mammary artery, and radial artery conduits—have been shown through various observational studies and meta-analyses to have superior outcomes compared with a SAG strategy.^6,7,8,9,10,11^ However, there remains a paucity of data demonstrating the association of preoperative ischemic heart failure with reduced ejection fraction with long-term outcomes when patients undergo revascularization by either SAG or MAG procedural strategies. Using a binational Australian and New Zealand cardiac surgical registry with long-term mortality data, we assessed the long-term survival difference of MAG vs SAG among patients stratified by preprocedural left ventricular ejection fraction (LVEF). In addition, we investigated whether the exclusion of SVG from MAG procedures (total arterial revascularization [TAR]) would be associated with any further survival advantages.

## Methods

### Study Design

Individuals undergoing primary isolated CABG at 59 cardiac institutions between June 1, 2001, and January 31, 2020, were identified via the Australian & New Zealand Society of Cardiac & Thoracic Surgeons (ANZSCTS) Cardiac Surgery Database, a centralized registry that functions under predetermined privacy regulations. The binational collection of data contains patient demographic, comorbidity, and procedural information and is regularly verified and linked with administrative datasets, including the National Death Index, to determine long-term mortality status. The registry, established to support systematic health care quality assurance and collaborative surgical research, operates on an approved opt-out model of participation with an overarching national ethics approval granted by the Alfred Health Institutional Ethics Review Board and the Melbourne Health Institutional Ethics Review Board. Additionally, local project-specific approval was obtained from the Melbourne Health Institutional Ethics Review Board with a waiver of individual patient consent due to the study’s retrospective design and the use of deidentified, encrypted data. The study results were reported in strict accordance with the Strengthening the Reporting of Observational Studies in Epidemiology (STROBE) reporting guideline.

Data entered into the ANZSCTS Cardiac Surgery Database undergo rigorous validation using predefined variables. The data management center ensures completeness and conducts quality assessments before reporting. Periodic on-site audits compare key data fields with original medical records to generate a research dataset.

### Patient Selection

**Figure 1.**
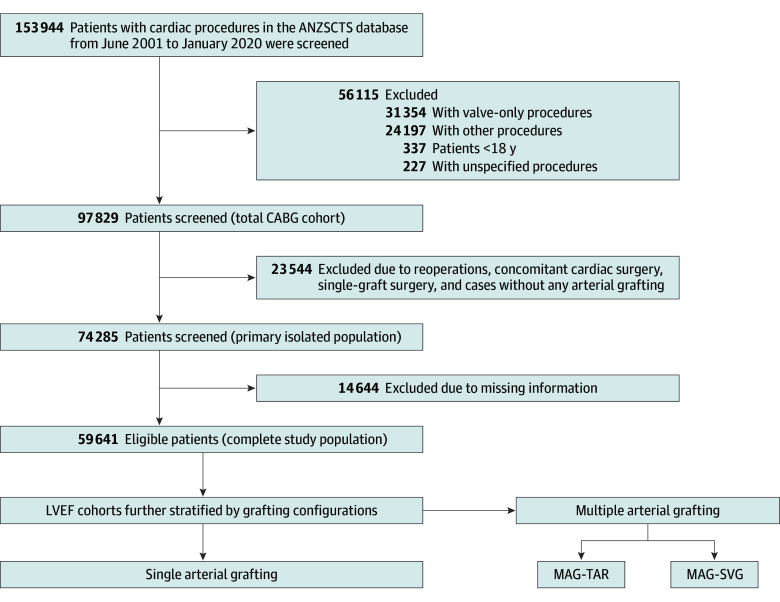
Patient Selection Process Comparisons among grafting strategies were conducted across individual patient cohorts with different degrees of left ventricular ejection dysfunction. ANZSCTS indicates the Australian & New Zealand Society of Cardiac & Thoracic Surgeons; CABG, coronary artery bypass grafting; LVEF, left ventricular ejection fraction; MAG-SVG, multiple arterial grafting–saphenous vein graft; and MAG-TAR, multiple arterial grafting–total arterial revascularization.

Patients who underwent primary isolated CABG with 2 or more grafts (anastomoses) were selected. Excluded from the study were nonadults (aged <18 years), cases of reoperations, concurrent or prior cardiac revascularization procedures, single-graft surgical procedures, and instances in which arterial grafting was completely absent. Patients with missing information were also excluded (Figure 1).

### Study Outcome

The primary end point assessed was long-term all-cause mortality, defined as the duration from the date of the index operation until the occurrence of the event or the censoring of the National Death Index linkage at the end of 2018. The median postoperative follow-up duration for the survival end point was 5.0 years (IQR, 2.3-8.6 years), and the maximum follow-up duration was 17.9 years. Institutional mortality records documented by participating hospitals were also included in the analysis if the event occurred after the censor date. Patients were stratified into 4 cohorts based on their LVEF: normal (>60%), mildly impaired (46%-60%), moderately impaired (30%-45%), and severely impaired (<30%).

### Statistical Analysis

A complete-case, population-based retrospective cohort analysis was conducted in September 2024 with continuous variables presented as median (IQR) or mean (SD) values, depending on the normality distribution, and categorical variables as frequency counts and percentages. Patients with missing information were excluded from quantitative analysis. Statistical significance was determined by 2-tailed *P* < .05. All statistical analyses were conducted using R, version 4.0.5 (R Project for Statistical Computing) with the following packages: survival, survminer, dplyr, WeightIt, MatchIt, and cobalt. Analyses were conducted per anastomosis, with individual sequential grafts considered as separate grafts. Analytical steps are summarized in eFigure 1 in Supplement 1.

#### Primary Analyses

Inverse probability treatment weighting (IPTW) was performed by using propensity scores to facilitate all comparisons between MAG and SAG, with marginal stabilization to ensure a balanced distribution of baseline characteristics. A detailed list of variables used in the propensity score calculation is in the eMethods in Supplement 1, which applied to all subsequent analyses. The absolute standardized mean differences (SMDs) were assessed before and after weighting as part of balance diagnostics; an observed difference of less than 10% typically indicated an equitable covariate distribution across comparative groups. We also generated love plots to assess the SMDs and histograms to examine propensity score distributions, providing a comprehensive visual representation of the balance achieved after adjustment. In individual time-to-event analyses of the 4 LVEF subgroups, we used an IPTW Cox proportional hazards regression model to compare the long-term survival of MAG vs SAG. Weighted Schoenfeld residuals were used to verify proportionality assumptions. Finally, the ejection fraction classification was introduced as an interaction term in a multivariable Cox proportional hazards regression analysis of the overall population, which included patients from all 4 LVEF subgroups, controlling for the same set of propensity score variables.

#### Secondary Analyses

Additional investigations were conducted to investigate any potential subgroup treatment effect from TAR for patients with MAG and differential ejection fraction. These patients with MAG were further stratified on the basis of SVG use, where we compared adjusted patients with MAG-TAR against those receiving any SVG (MAG-SVG) in all 4 LVEF categories. The same variables were adjusted (eMethods in Supplement 1).

#### Sensitivity Analysis

A series of alternative baseline adjustment methods were applied to the primary investigations to assess the robustness of our findings. Specifically, we (1) removed the stabilization process from IPTW and (2) performed 1:1 greedy propensity score matching with a caliper width of 0.2 SDs of the logit of the propensity scores, without replacement, accounting for all variables (eMethods in Supplement 1). After the propensity score matching, sandwich-type robust variance estimators were used to adjust for the random clustering effect within individual patient pairs.

## Results

A total of 153 944 patients were identified from the ANZSCTS Cardiac Surgery Database as having undergone primary isolated coronary bypass surgery between June 2001 and January 2020. Of these, 59 641 patients (mean [SD] age at the time of surgery, 65.8 [10.2] years; 48 321 men [81.0%] and 11 320 women [19.0%]) met the predefined eligibility criteria, undergoing primary isolated CABG with at least 2 grafts (Figure 1). The overall utilization rate of MAG for the study cohort was 58.8% (35 077 of 59 641 patients). A total of 29 891 patients (50.1%) had normal LVEF (>60%), 18 980 patients (31.8%) had mild LVEF impairment (46%-60%), 8640 patients (14.5%) had moderate LVEF impairment (30%-45%), and 2130 patients (3.6%) had severe LVEF impairment (<30%). The median postoperative follow-up duration was 5.0 years (IQR, 2.3-8.6 years), and the maximum follow-up duration was 17.9 years.

At baseline, patients with severe left ventricular (LV) dysfunction, compared with those in the normal LVEF cohort, had increased rates of cigarette smoking, diabetes, dialysis-dependent kidney failure, peripheral vascular disease, prior myocardial infarction, congestive heart failure, respiratory disease, and triple-vessel disease, as well as an urgent indication for revascularization procedures (Table). Furthermore, those with severe LV dysfunction were more likely to have undergone on-pump surgery and received a greater number of distal anastomoses. Patients with preserved LVEF exhibited a higher prevalence of hypercholesterolemia and elective procedures. The utilization rate of MAG incrementally decreased as LVEF worsened, from 61.6% (18 399 of 29 891) for patients with no LV impairment to 57.8% (10 979 of 18 980) for patients with mild impairment, 54.3% (4694 of 8640) for patients with moderate impairment, and 47.2% (1005 of 2130) for patients with severe impairment. The proportionality assumption was not violated in any model (eTable 1 in Supplement 1).

**Table.** Preoperative Demographic Characteristics According to LVEF Classifications

Characteristic	Patients, No. (%)			
	Normal (LVEF >60%) (n = 29 891)	Mild impairment (LVEF 46%-60%) (n = 18 980)	Moderate impairment (LVEF 30%-45%) (n = 8640)	Severe impairment (LVEF <30%) (n = 2130)
Multiarterial grafting	18 399 (61.6)	10 979 (57.8)	4694 (54.3)	1005 (47.2)
Age, mean (SD), y	65.9 (9.9)	65.9 (10.3)	65.9 (10.6)	65.1 (10.7)
Sex				
Male	23 731 (79.4)	15 592 (82.1)	7185 (83.2)	1813 (85.1)
Female	6160 (20.6)	3388 (17.9)	1455 (16.8)	317 (14.9)
BMI, mean (SD)	29.0 (7.7)	29.1 (7.8)	29.0 (7.7)	28.4 (10.8)
Smoking history	18 521 (62.0)	12 510 (65.9)	6024 (69.7)	1535 (72.1)
Diabetes	10 159 (34.0)	6934 (36.5)	3718 (43.0)	1050 (49.3)
Hypercholesterolemia	24 426 (81.7)	15 433 (81.3)	6807 (78.8)	1650 (77.5)
Creatinine, mean (SD), mg/dL	1.1 (0.8)	1.1 (0.9)	1.2 (1.1)	1.3 (1.0)
Dialysis	261 (0.9)	310 (1.6)	204 (2.4)	56 (2.6)
Hypertension	23 792 (79.6)	15 147 (79.8)	6894 (79.8)	1625 (76.3)
Cerebrovascular event	2676 (9.0)	1854 (9.8)	1063 (12.3)	249 (11.7)
Peripheral vascular disease	2637 (8.8)	2072 (10.9)	1261 (14.6)	315 (14.8)
Respiratory disease	3063 (10.2)	2275 (12.0)	1333 (15.4)	336 (15.8)
Myocardial infarction	12 132 (40.6)	11 102 (58.5)	6570 (76.0)	1668 (78.3)
Congestive heart failure	2068 (6.9)	1788 (9.4)	2298 (26.6)	1106 (51.9)
CCS classification ≥3	10 832 (36.2)	7783 (41.0)	3794 (43.9)	970 (45.5)
NYHA classification ≥3	4208 (14.1)	3045 (16.0)	2262 (26.2)	1001 (47.0)
Cardiogenic shock	78 (0.3)	129 (0.7)	256 (3.0)	191 (9.0)
Resuscitation	70 (0.2)	82 (0.4)	118 (1.4)	85 (4.0)
Arrhythmia	1771 (5.9)	1632 (8.6)	1188 (13.8)	415 (19.5)
Left main coronary artery disease	8242 (27.6)	4922 (25.9)	1333 (15.4)	639 (30.0)
No. of diseased territories, mean (SD)	2.7 (0.5)	2.7 (0.5)	2.8 (0.5)	2.8 (0.4)
Single-vessel disease	650 (2.2)	306 (1.6)	100 (1.2)	17 (0.8)
Double-vessel disease	7804 (26.1)	4210 (22.2)	1497 (17.3)	330 (15.5)
Triple-vessel disease	21 297 (71.2)	14 387 (75.8)	7009 (81.1)	1773 (83.2)
Perioperative medications				
Inotropes	445 (1.5)	251 (1.3)	242 (2.8)	217 (10.2)
Nitroglycerin	1540 (5.2)	1115 (5.9)	682 (7.9)	222 (10.4)
Anticoagulants	5769 (19.3)	4371 (23.0)	2420 (28.0)	730 (34.3)
Corticosteroids	395 (1.3)	264 (1.4)	127 (1.5)	43 (2.0)
LVEF measurement method				
Angiography	13 078 (43.8)	7124 (37.5)	2944 (34.1)	608 (28.5)
Radionuclide	208 (0.7)	166 (0.9)	165 (1.9)	111 (5.2)
Echocardiography	16 294 (54.5)	11 573 (61.0)	5454 (63.1)	1371 (64.4)
MRI	7 (0.0)	13 (0.1)	39 (0.5)	27 (1.3)
Elective	20 231 (67.7)	11 391 (60.0)	4678 (54.1)	1021 (47.9)
Urgent	9080 (30.4)	7057 (37.2)	3476 (40.2)	875 (41.1)
No. of grafts, mean (SD)	3.3 (1.0)	3.3 (0.9)	3.4 (1.0)	3.5 (1.0)
On-pump surgery	28 001 (93.7)	17 571 (92.6)	8118 (93.6)	2033 (95.4)

Abbreviations: BMI, body mass index (calculated as weight in kilograms divided by height in meters squared); CCS, Canadian Cardiovascular Society; LVEF, left ventricular ejection fraction; MRI, magnetic resonance imaging; NYHA, New York Heart Association.

SI conversion factor: To convert creatinine to micromoles per liter, multiply by 88.4.

### Primary Analyses

**Figure 2.**
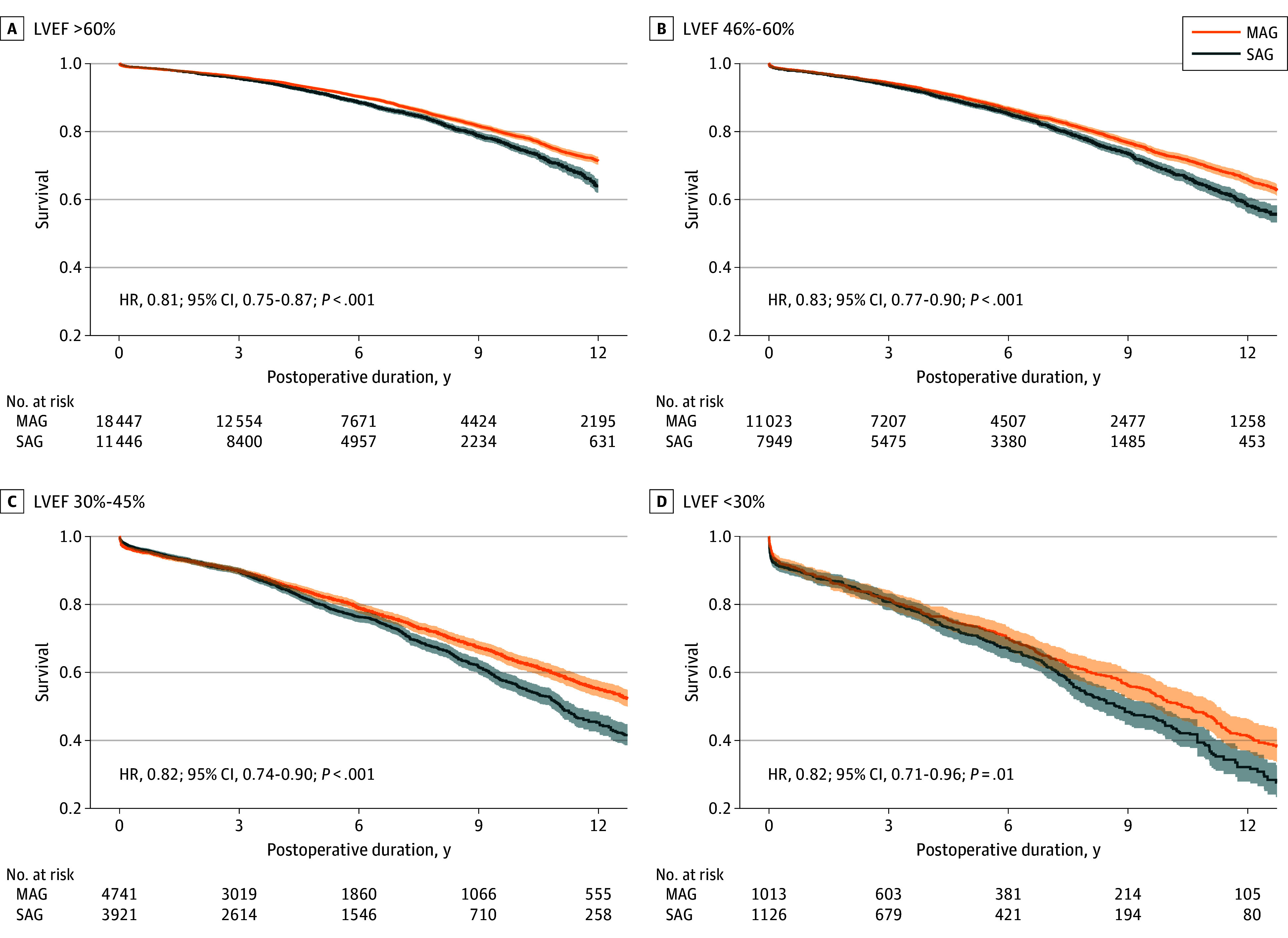
Adjusted Kaplan-Meier Survival Curves for Multiple Arterial Grafting (MAG) vs Single Arterial Grafting (SAG) Stratified by Left Ventricular Ejection Fraction (LVEF) Adjustment of Kaplan-Meier survival estimations was achieved through inverse probability treatment weighting, creating a pseudopopulation for balanced comparison, which may result in the number of patients at risk differing from the absolute sample size. MAG patients received more than 1 arterial conduit during operation, whereas SAG patients received only 1 arterial conduit. Shaded areas indicate 95% CIs. HR indicates hazard ratio.

The IPTW risk adjustment reduced all SMDs to below 0.1 (eTables 2-5 in Supplement 1), ensuring balanced comparisons. Plots of the propensity score distributions (eFigures 2-5 in Supplement 1) and the SMDs (eFigures 6-9 in Supplement 1) before and after adjustment are provided in Supplement 1. For the normal LVEF group, patients treated with MAG compared with SAG had a 19.0% relative reduction in all-cause mortality (hazard ratio [HR], 0.81; 95% CI, 0.75-0.87; *P* < .001). Significant survival advantages for the MAG group were also observed among patients with mild LV impairment (HR, 0.83; 95% CI, 0.77-0.90; *P* < .001), moderate LV impairment (HR, 0.82; 95% CI, 0.74-0.90; *P* < .001), and severe LV impairment (HR, 0.82; 95% CI, 0.71-0.96; *P* = .01). A multivariable Cox proportional hazards regression interaction term analysis of the combined population indicated that differential LVEF did not (*P* = .75) have a significant subgroup association with the survival comparison between MAG and SAG. Associated Kaplan-Meier visualizations of the survival differences are presented in Figure 2.

### Secondary Analyses

**Figure 3.**
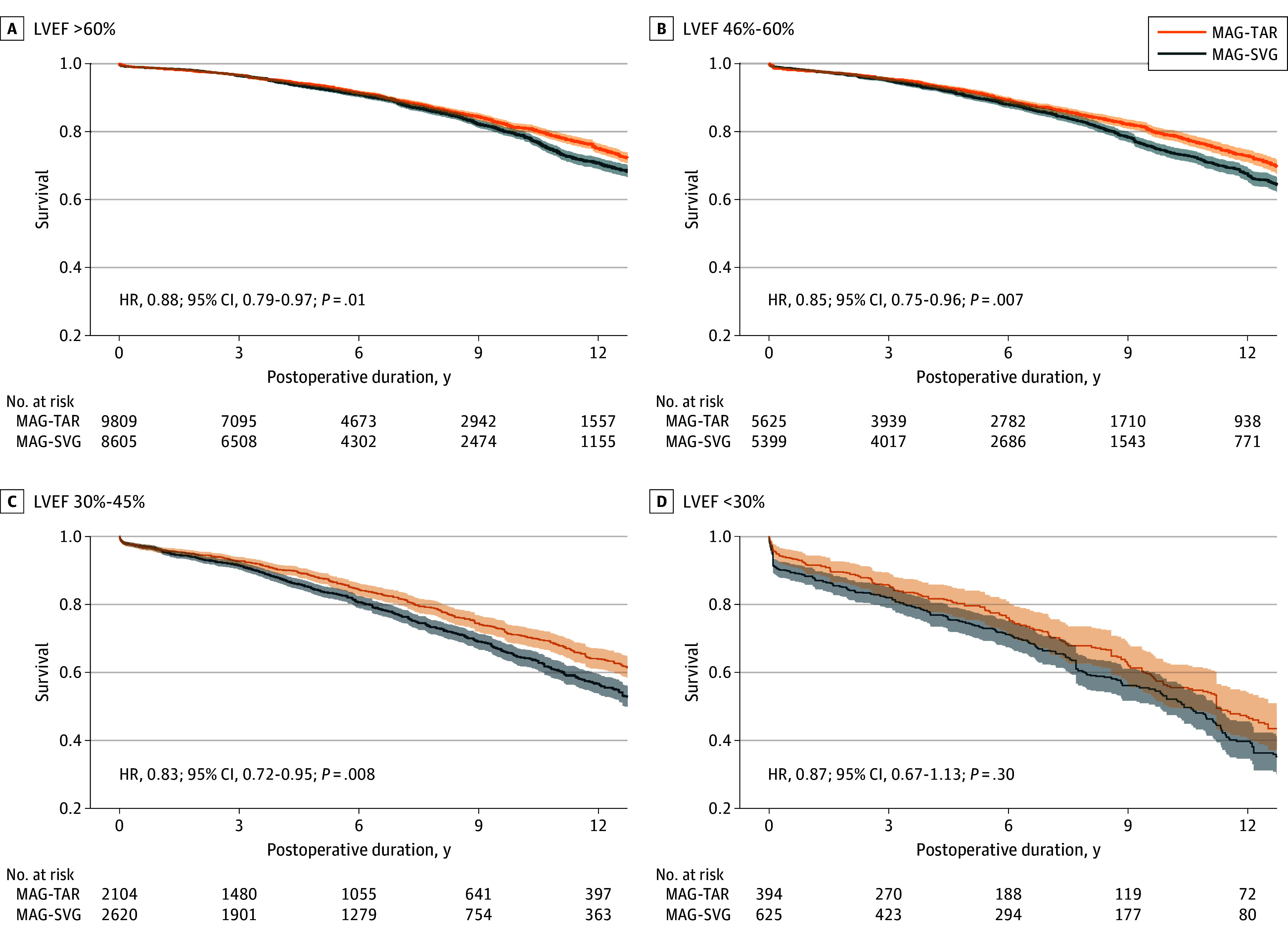
Adjusted Kaplan-Meier Survival Curves for Multiple Arterial Grafting (MAG) Procedures With vs Without Saphenous Vein Graft (SVG) Stratified by Left Ventricular Ejection Fraction (LVEF) Adjustment of Kaplan-Meier survival estimations was achieved through inverse probability treatment weighting, creating a pseudopopulation for balanced comparison, which may result in the number of patients at risk differing from the absolute sample size. The MAG-TAR (multiple arterial grafting–total arterial revascularization) patient group received multiple arterial grafts without any supplementary saphenous vein grafts, whereas the MAG-SVG group received multiple arterial grafts with at least 1 saphenous vein graft. Shaded areas indicate 95% CIs. HR indicates hazard ratio.

For prespecified comparisons within the MAG cohort, patients without an SVG (MAG-TAR) showed significantly improved long-term survival compared with those receiving any venous grafting (MAG-SVG) across the spectrum of normal LVEF (HR, 0.88; 95% CI, 0.79-0.97; *P* = .01), mildly impaired LVEF (HR, 0.85; 95% CI, 0.75-0.96; *P* = .007), and moderately impaired LVEF (HR, 0.83; 95% CI, 0.72-0.95; *P* = .008). However, the survival benefit was not associated with MAG-TAR among patients with severely impaired LVEF (HR, 0.87; 95% CI, 0.67-1.13; *P* = .30). Associated Kaplan-Meier survival function curves are presented in Figure 3. Detailed patient demographic characteristics and SMDs of each comparison are presented in eTables 6 to 9 in Supplement 1. eFigures 10 to 17 in Supplement 1 demonstrate the propensity score distributions and the absolute SMDs before and after adjustment.

### Sensitivity Analyses

All groups from sensitivity analyses demonstrated well-balanced baseline covariates (eTables 10-17 in Supplement 1). After removing stabilization from the IPTW algorithm, we observed consistent survival improvement associated with MAG compared with SAG procedures for patients with no LV impairment (HR, 0.81; 95% CI, 0.75-0.87; *P* < .001), mild LV impairment (HR, 0.83; 95% CI, 0.77-0.90; *P* < .001), moderate LV impairment (HR, 0.81; 95% CI, 0.74-0.90; *P* < .001), and severe LV impairment (HR, 0.82; 95% CI, 0.71-0.96; *P* = .01).

The propensity score matching algorithm produced 10 891 matched pairs for the normal LVEF cohort (HR, 0.79; 95% CI, 0.73-0.85; *P* < .001), 7311 pairs for the mildly impaired LVEF cohort (HR, 0.77; 95% CI, 0.71-0.84; *P* < .001), 3366 pairs for the moderately impaired LVEF cohort (HR, 0.80; 95% CI, 0.73-0.88; *P* < .001), and 765 pairs for the severely impaired LVEF cohort (HR, 0.82; 95% CI, 0.70-0.96; *P* = .01). Again, MAG had significantly superior long-term survival outcomes compared with SAG across all levels of LV impairment, corroborating the findings from our primary investigations.

## Discussion

In the present study, which uses a large binational Australia and New Zealand multicenter surgical dataset, we demonstrated that the use of MAG was associated with a significant improvement in long-term survival compared with SAG among patients undergoing CABG, regardless of their preoperative LV systolic function. To our knowledge, this analysis represents one of the largest and most comprehensive studies globally on this research question, and it is the first to establish an association between the use of MAG, despite the inherent technical complexities, and enhanced long-term survival among patients with varying degrees of LVEF, from preserved to severely reduced at the time of revascularization. This observation was further substantiated by our interaction term analysis, confirming that LVEF levels did not influence the survival benefit associated with MAG. TAR emerged as the superior subset within MAG procedures, associated with greater survival benefits compared with MAG configurations that included any SVG across all LVEF subgroups except in cases of severe LV impairment.

Although numerous studies^9,10,11,12,13^ have documented substantial survival benefits associated with MAG vs SAG procedures, very few studies specifically targeted populations with impaired ejection fraction and reported conflicting findings. In a propensity score–matched investigation of 1641 patients with mild to moderate ejection impairment, MAG was not significantly associated with reduced midterm mortality compared with SAG (12.2% vs 18.4%; HR, 0.77; 95% CI, 0.47-1.24; *P* = .29).^14^ Conversely, another study of 20 076 patients conducted by Pu et al^15^ demonstrated similar relative reductions as those observed in our study for long-term mortality risk among patients undergoing MAG vs SAG with normal LVEF (HR, 0.77; 95% CI, 0.68-0.89) and moderately impaired LVEF (HR, 0.77; 95% CI, 0.66-0.90). However, this survival advantage was not extended to patients with LVEF below 35% (HR, 1.12; 95% CI, 0.87-1.45), in contrast to our findings. The absence of a survival difference may be associated with the limited sample size relative to our corresponding patient subgroup with severely impaired LVEF.

Moreover, this British Columbia registry^15^ also reported a progressively decreasing utilization rate of MAG with increasing LVEF impairment, a trend that aligns with our national practice patterns. The surgical preference may be associated with the perceived technical challenges and longer operative times associated with arterial harvesting and revascularization, as well as a conservative approach by surgeons when operating on patients at high risk.^16^ In addition, historical uncertainty regarding the long-term benefits associated with MAG for patients with various degrees of LVEF impairment has contributed to a knowledge gap, limiting its broader adoption. Our current investigation addresses this concern comprehensively. The inclusion of the interaction term in our Cox proportional hazards regression model was prespecified during the study design phase to evaluate whether varying degrees of LVEF dysfunction would influence the long-term survival benefits associated with MAG. The interaction model revealed that the survival difference between MAG vs SAG remained consistent and unaffected across any preoperative LVEF levels. This approach provided a robust framework for interpreting our stratified analyses and offered unique insights into the association between LVEF and the treatment effect of MAG.

Another important observation from our current analyses was that the inclusion of SVG appeared to compromise the survival benefits typically associated with MAG procedures when the LVEF was greater than 30%. This is a novel finding, as no prior studies have conducted similar analyses with LVEF stratifications to our knowledge. The Arterial Revascularization Trial (ART)^17^ did not identify a significant 10-year survival difference between bilateral internal mammary artery grafting and single internal mammary artery grafting, a result largely associated with a high crossover rate and the outdated assumption that radial artery grafts perform similarly to SVG. The bilateral internal mammary artery grafting cohort in the ART had no restrictions on vein graft use, which may have confounded the true underlying survival benefits associated with MAG. As indicated in the authors’ post hoc analysis^18^ comparing MAG and TAR against SAG, TAR was associated with the lowest risk of late mortality (HR, 0.68; 95% CI, 0.48-0.96; *P* = .03), consistent with our observations. In other words, TAR may represent a superior MAG configuration, offering the greatest survival benefit and potentially serving as the primary factor associated with the improved survival observed with MAG. The recently initiated randomized multicenter Total Arterial Revascularization Trial in Australia is expected to offer valuable insights, with a comprehensive assessment of both angiographic and clinical outcomes comparing TAR and non-TAR procedures.^19^ The lack of significance observed in our comparison between MAG-TAR and MAG-SVG for patients with LVEF less than 30% is likely associated with statistical limitations. In particular, the relatively small sample size in this subgroup (n = 1005), which was further reduced by 2 rounds of stratification, increases the risk of a type II error. This limitation may obscure potential differences in survival outcomes. Addressing this issue will require future studies with adequately powered, selected cohorts to validate these findings and provide clearer guidance for clinical decision-making in this specific population.

Left ventricular ejection fraction is a widely used index of LV contractility that provides an important prognostic assessment for cardiovascular disease by reflecting the percentage of blood volume ejected from the ventricle during systole relative to the volume present at the end of diastole.^20^ Despite its clinical utility, there is no universally agreed-on threshold for a normal LVEF value due to significant variations depending on patient demographics and the methods used for measurement.^21^ All patients in the current dataset had their ejection fraction quantitatively measured using angiography, radionuclide imaging, echocardiography, or magnetic resonance imaging. Recognizing that different imaging modalities can produce substantially variable estimations,^22^ we thought it was imperative to incorporate an adjustment for measurement methods into our weighting algorithm to further enhance the robustness of our analysis.

Change in clinical practice typically occurs gradually and is often driven by local champions of innovation. It is advisable for surgical units to adopt a phased approach when implementing MAG and TAR. For instance, starting with the increased use of the radial artery as a graft while maintaining conventional techniques allows teams to gain familiarity and confidence before progressing to more advanced methods, such as sequential and composite grafting. This gradual transition also facilitates adjustments to perioperative management protocols as needed. The resources and operating times required for TAR are comparable with those of conventional techniques, but the absence of comprehensive training materials remains a significant barrier to implementing this change.

### Limitations and Strengths

This study has some limitations. The lack of survival benefit observed in the MAG-TAR vs MAG-SVG groups for patients with severe LV impairment is likely attributable to a type II error resulting from insufficient statistical power rather than a true biological effect. In addition, we recognize the potential influence of residual confounding and treatment allocation biases inherent to the retrospective study design, even with rigorous adjustments, warranting cautious interpretation of the findings. Graft patency, other nonfatal outcomes, and site-specific procedural preferences were not captured in our current dataset. Nonetheless, the study’s strengths include its robust methods, extensive dataset, and detailed subgroup analyses, which provide valuable insights into arterial revascularization outcomes. Future prospective studies with adequately powered and targeted cohorts are crucial to validate these findings and further inform clinical decision-making in this population.

## Conclusions

In this retrospective cohort study using a binational cardiac surgery database, MAG in CABG significantly reduced the risk of long-term all-cause mortality compared with SAG regardless of the level of preoperative LVEF. The survival advantage was greatest when TAR was achieved, particularly among patients with preserved LVEF. Because most coronary surgery practice continues to use single arterial grafting, consideration to alter grafting strategy to multiarterial procedures may be indicated.

## Notes

Supplement 1. **eMethods.** Preoperative, Operative and Demographic Variables for Propensity Score Calculation and Comprehensive Adjustment **eTable 1.** Verification of the Proportional Hazards Assumption Using Schoenfeld Residuals **eTable 2.** Patient Demographics of MAG vs SAG Cohorts With LVEF >60% **eTable 3.** Patient Demographics of MAG vs SAG Cohorts With LVEF 46-60% **eTable 4.** Patient Demographics of MAG vs SAG Cohorts With LVEF 30-45% **eTable 5.** Patient Demographics of MAG vs SAG Cohorts With LVEF <30% **eTable 6.** Patient Demographics of MAG-TAR and MAG-SVG Cohorts With LVEF >60% **eTable 7.** Patient Demographics of MAG-TAR and MAG-SVG Cohorts With LVEF 46-60% **eTable 8.** Patient Demographics of MAG-TAR and MAG-SVG Cohorts With LVEF 30-45% **eTable 9.** Patient Demographics of MAG-TAR and MAG-SVG Cohorts With LVEF <30% **eTable 10.** Patient Demographics of MAG and SVG Cohorts in Sensitivity Analysis Without Stabilization During Inverse Probability Weighting (LVEF >60%) **eTable 11.** Patient Demographics of MAG and SVG Cohorts in Sensitivity Analysis Without Stabilization During Inverse Probability Weighting (LVEF 46-60%) **eTable 12.** Patient Demographics of MAG and SVG Cohorts in Sensitivity Analysis Without Stabilization During Inverse Probability Weighting (LVEF 30-45%) **eTable 13.** Patient Demographics of MAG and SVG Cohorts in Sensitivity Analysis Without Stabilization During Inverse Probability Weighting (LVEF <30%) **eTable 14.** Patient Demographics of MAG and SVG Cohorts Before and After Propensity Score Matching (LVEF >60%) **eTable 15.** Patient Demographics of MAG and SVG Cohorts Before and After Propensity Score Matching (LVEF 46-60%) **eTable 16.** Patient Demographics of MAG and SVG Cohorts Before and After Propensity Score Matching (LVEF 30-45%) **eTable 17.** Patient Demographics of MAG and SVG Cohorts Before and After Propensity Score Matching (LVEF <30%) **eFigure 1.** Summary of the Analytical Steps **eFigure 2.** Histogram of Propensity Score Distribution in MAG vs SAG Subgroup (LVEF >60%) Before and After Inverse Probability Weighting **eFigure 3.** Histogram of Propensity Score Distribution in MAG vs SAG Subgroup (LVEF 46-60%) Before and After Inverse Probability Weighting **eFigure 4.** Histogram of Propensity Score Distribution in MAG vs SAG Subgroup (LVEF 30-45%) Before and After Inverse Probability Weighting **eFigure 5.** Histogram of Propensity Score Distribution in MAG vs SAG Subgroup (LVEF <30%) Before and After Inverse Probability Weighting **eFigure 6.** Absolute Standardized Mean Differences of Covariates Before and After Adjustment in the MAG vs SAG Comparison (LVEF >60%) **eFigure 7.** Absolute Standardized Mean Differences of Covariates Before and After Adjustment in the MAG vs SAG Comparison (LVEF 46-60%) **eFigure 8.** Absolute Standardized Mean Differences of Covariates Before and After Adjustment in the MAG vs SAG Comparison (LVEF 30-45%) **eFigure 9.** Absolute Standardized Mean Differences of Covariates Before and After Adjustment in the MAG vs SAG Comparison (LVEF <30%) **eFigure 10.** Histogram of Propensity Score Distribution in MAG-TAR vs MAG-SVG Subgroup (LVEF >60%) Before and After Inverse Probability Weighting **eFigure 11.** Histogram of Propensity Score Distribution in MAG-TAR vs MAG-SVG Subgroup (LVEF 46-60%) Before and After Inverse Probability Weighting **eFigure 12.** Histogram of Propensity Score Distribution in MAG-TAR vs MAG-SVG Subgroup (LVEF 30-45%) Before and After Inverse Probability Weighting **eFigure 13.** Histogram of Propensity Score Distribution in MAG-TAR vs MAG-SVG Subgroup (LVEF <30%) Before and After Inverse Probability Weighting **eFigure 14.** Absolute Standardized Mean Differences of Covariates Before and After Adjustment in the MAG-TAR vs MAG-SVG Comparison (LVEF >60%) **eFigure 15.** Absolute Standardized Mean Differences of Covariates Before and After Adjustment in the MAG-TAR vs MAG-SVG Comparison (LVEF 46-60%) **eFigure 16.** Absolute Standardized Mean Differences of Covariates Before and After Adjustment in the MAG-TAR vs MAG-SVG Comparison (LVEF 30-45%) **eFigure 17.** Absolute Standardized Mean Differences of Covariates Before and After Adjustment in the MAG-TAR vs MAG-SVG Comparison (LVEF <30%)

Supplement 2.
Data Sharing Statement
