# Supplementary material for: Survival Outcomes After Multiple vs Single Arterial Grafting Among Patients With Reduced Ejection Fraction
Source: JAMA Netw Open. 2025 Apr 10;8(4):e254508. doi: 10.1001/jamanetworkopen.2025.4508 (PMC11986767; doi:10.1001/jamanetworkopen.2025.4508)
Supplement: Supplement 1. — eMethods. Preoperative, Operative and Demographic Variables for Propensity Score Calculation and Comprehensive Adjustment eTable 1. Verification of the Proportional Hazards Assumption Using Schoenfeld Residuals eTable 2. Patient Demographics of MAG vs SAG Cohorts With LVEF >60% eTable 3. Patient Demographics of MAG vs SAG Cohorts With LVEF 46-60% eTable 4. Patient Demographics of MAG vs SAG Cohorts With LVEF 30-45% eTable 5. Patient Demographics of MAG vs SAG Cohorts With LVEF <30% eTable 6. Patient Demographics of MAG-TAR and MAG-SVG Cohorts With LVEF >60% eTable 7. Patient Demographics of MAG-TAR and MAG-SVG Cohorts With LVEF 46-60% eTable 8. Patient Demographics of MAG-TAR and MAG-SVG Cohorts With LVEF 30-45% eTable 9. Patient Demographics of MAG-TAR and MAG-SVG Cohorts With LVEF <30% eTable 10. Patient Demographics of MAG and SVG Cohorts in Sensitivity Analysis Without Stabilization During Inverse Probability Weighting (LVEF >60%) eTable 11. Patient Demographics of MAG and SVG Cohorts in Sensitivity Analysis Without Stabilization During Inverse Probability Weighting (LVEF 46-60%) eTable 12. Patient Demographics of MAG and SVG Cohorts in Sensitivity Analysis Without Stabilization During Inverse Probability Weighting (LVEF 30-45%) eTable 13. Patient Demographics of MAG and SVG Cohorts in Sensitivity Analysis Without Stabilization During Inverse Probability Weighting (LVEF <30%) eTable 14. Patient Demographics of MAG and SVG Cohorts Before and After Propensity Score Matching (LVEF >60%) eTable 15. Patient Demographics of MAG and SVG Cohorts Before and After Propensity Score Matching (LVEF 46-60%) eTable 16. Patient Demographics of MAG and SVG Cohorts Before and After Propensity Score Matching (LVEF 30-45%) eTable 17. Patient Demographics of MAG and SVG Cohorts Before and After Propensity Score Matching (LVEF <30%) eFigure 1. Summary of the Analytical Steps eFigure 2. Histogram of Propensity Score Distribution in MAG vs SAG Subgroup (LVEF >60%) Before and After Inve [file jamanetwopen-e254508-s001.pdf]

Supplementary Online Content

Ren J, Bloom JE, Chan W, et al. Survival outcomes after multiple vs single arterial grafting among patients with reduced ejection fraction. *JAMA Netw Open.* 2025;8(4):e254508. doi:10.1001/jamanetworkopen.2025.4508

eMethods. Preoperative, Operative and Demographic Variables for Propensity Score Calculation and Comprehensive Adjustment
eTable 1. Verification of the Proportional Hazards Assumption Using Schoenfeld Residuals
eTable 2. Patient Demographics of MAG vs SAG Cohorts With LVEF >60%
eTable 3. Patient Demographics of MAG vs SAG Cohorts With LVEF 46-60%
eTable 4. Patient Demographics of MAG vs SAG Cohorts With LVEF 30-45%
eTable 5. Patient Demographics of MAG vs SAG Cohorts With LVEF <30%
eTable 6. Patient Demographics of MAG-TAR and MAG-SVG Cohorts With LVEF >60%
eTable 7. Patient Demographics of MAG-TAR and MAG-SVG Cohorts With LVEF 46-60%
eTable 8. Patient Demographics of MAG-TAR and MAG-SVG Cohorts With LVEF 30-45%
eTable 9. Patient Demographics of MAG-TAR and MAG-SVG Cohorts With LVEF <30%
eTable 10. Patient Demographics of MAG and SVG Cohorts in Sensitivity Analysis Without Stabilization During Inverse Probability Weighting (LVEF >60%)
eTable 11. Patient Demographics of MAG and SVG Cohorts in Sensitivity Analysis Without Stabilization During Inverse Probability Weighting (LVEF 46-60%)
eTable 12. Patient Demographics of MAG and SVG Cohorts in Sensitivity Analysis Without Stabilization During Inverse Probability Weighting (LVEF 30-45%)
eTable 13. Patient Demographics of MAG and SVG Cohorts in Sensitivity Analysis Without Stabilization During Inverse Probability Weighting (LVEF <30%)
eTable 14. Patient Demographics of MAG and SVG Cohorts Before and After Propensity Score Matching (LVEF >60%)
eTable 15. Patient Demographics of MAG and SVG Cohorts Before and After Propensity Score Matching (LVEF 46-60%)
eTable 16. Patient Demographics of MAG and SVG Cohorts Before and After Propensity Score Matching (LVEF 30-45%)
eTable 17. Patient Demographics of MAG and SVG Cohorts Before and After Propensity Score Matching (LVEF <30%)
eFigure 1. Summary of the Analytical Steps
eFigure 2. Histogram of Propensity Score Distribution in MAG vs SAG Subgroup (LVEF >60%) Before and After Inverse Probability Weighting

© 2025 Ren J et al. *JAMA Network Open.*

eFigure 3. Histogram of Propensity Score Distribution in MAG vs SAG Subgroup (LVEF 46-60%) Before and After Inverse Probability Weighting
eFigure 4. Histogram of Propensity Score Distribution in MAG vs SAG Subgroup (LVEF 30-45%) Before and After Inverse Probability Weighting
eFigure 5. Histogram of Propensity Score Distribution in MAG vs SAG Subgroup (LVEF <30%) Before and After Inverse Probability Weighting
eFigure 6. Absolute Standardized Mean Differences of Covariates Before and After Adjustment in the MAG vs SAG Comparison (LVEF >60%)
eFigure 7. Absolute Standardized Mean Differences of Covariates Before and After Adjustment in the MAG vs SAG Comparison (LVEF 46-60%)
eFigure 8. Absolute Standardized Mean Differences of Covariates Before and After Adjustment in the MAG vs SAG Comparison (LVEF 30-45%)
eFigure 9. Absolute Standardized Mean Differences of Covariates Before and After Adjustment in the MAG vs SAG Comparison (LVEF <30%)
eFigure 10. Histogram of Propensity Score Distribution in MAG-TAR vs MAG-SVG Subgroup (LVEF >60%) Before and After Inverse Probability Weighting
eFigure 11. Histogram of Propensity Score Distribution in MAG-TAR vs MAG-SVG Subgroup (LVEF 46-60%) Before and After Inverse Probability Weighting
eFigure 12. Histogram of Propensity Score Distribution in MAG-TAR vs MAG-SVG Subgroup (LVEF 30-45%) Before and After Inverse Probability Weighting
eFigure 13. Histogram of Propensity Score Distribution in MAG-TAR vs MAG-SVG Subgroup (LVEF <30%) Before and After Inverse Probability Weighting
eFigure 14. Absolute Standardized Mean Differences of Covariates Before and After Adjustment in the MAG-TAR vs MAG-SVG Comparison (LVEF >60%)
eFigure 15. Absolute Standardized Mean Differences of Covariates Before and After Adjustment in the MAG-TAR vs MAG-SVG Comparison (LVEF 46-60%)
eFigure 16. Absolute Standardized Mean Differences of Covariates Before and After Adjustment in the MAG-TAR vs MAG-SVG Comparison (LVEF 30-45%)
eFigure 17. Absolute Standardized Mean Differences of Covariates Before and After Adjustment in the MAG-TAR vs MAG-SVG Comparison (LVEF <30%)

This supplementary material has been provided by the authors to give readers additional information about their work.

© 2025 Ren J et al. *JAMA Network Open.*

eMethods. Preoperative, Operative and Demographic Variables for Propensity Score Calculation and Comprehensive Adjustment

Through multivariable logistic regression models, propensity scores were prepared separately for each EF-stratified comparisons between MAG versus SAG, and for MAG without versus with SVG (MAG-TAR versus MAG-SVG). The implementation of propensity scores in all our adjustment algorithms accounted for age, smoking history, hypertension, hypercholesterolemia, diabetes mellitus, dialysis, cerebrovascular disease, peripheral vascular disease, chronic respiratory disease, preoperative myocardial infarction, arrhythmia, cardiogenic shock, resuscitation, New York Heart Association (NYHA) classification, Canadian Cardiovascular Society (CCS) classification, congestive heart failure, body mass index, creatinine level, left main disease, and the number of diseased territories. Operative details including the year of operation, number of grafts, minimally invasive procedures, on-pump surgery, and operative status were statistically adjusted. Measurement methods for LVEF were comprehensively captured and adjusted to ensure accuracy and consistency across the study. These methods included angiogram (left ventriculography performed during cardiac catheterization), radionuclide imaging (nuclear studies), echocardiography (both transthoracic and transesophageal), and magnetic resonance imaging (MRI). Additionally, preoperative administration of key therapies—such as inotropes, intravenous nitrates, anticoagulation therapy, and steroids on the day of surgery—was carefully documented and adjusted to account for their potential influence on outcomes.

© 2025 Ren J et al. *JAMA Network Open.*

eTable 1. Verification of the Proportional Hazards Assumption Using Schoenfeld Residuals

**Cox Regression Comparisons**	**Schoenfeld Residual P-Values**
MAG versus SAG	
LVEF >60% Group	0.13
LVEF 46-60% Group	0.23
LVEF 30-45% Group	0.27
LVEF 0-30% Group	0.62
MAG-TAR versus MAG-SVG	
LVEF >60% Group	0.05
LVEF 46-60% Group	0.57
LVEF 30-45% Group	0.86
LVEF 0-30% Group	0.76

MAG, multiple arterial grafting; SAG, single arterial grafting; TAR, total arterial revascularisation; SVG, saphenous vein graft; LVEF, left ventricular ejection fraction.

© 2025 Ren J et al. *JAMA Network Open.*

eTable 2. Patient Demographics of MAG vs SAG Cohorts With LVEF >60%

Characteristics<br>Patients, No. (%)	SAG	MAG	Unweighted SMD	Weighted SMD
Total sample size	11492	18399	-	-
Age, mean ± SD	67.1 ± 9.8	65.1 ± 10.0	0.196	0.006
Male	8876 (77.2)	14855 (80.7)	0.086	0.002
Body mass index	29.1 ± 8.6	29.0 ± 7.0	0.007	0.006
Smoking history	7254 (63.1)	11267 (61.2)	0.039	0.008
Diabetes	4267 (37.1)	5892 (32.0)	0.108	0.004
Hypercholesterolemia	9300 (81.7)	15036 (81.7)	0	0.010
Creatinine, mean ± SD, µmol/L	99.1 ± 80.3	92.2 ± 55.7	0.099	0.012
Dialysis	179 (1.6)	82 (0.5)	0.112	0.002
Hypertension	9459 (82.1)	14333 (77.9)	0.111	0
Cerebrovascular event	1143 (9.95)	1533 (8.3)	0.056	0.001
Peripheral vascular disease	1089 (9.5)	1548 (8.4)	0.037	0.004
Respiratory disease	1323 (11.5)	1740 (9.5)	0.067	0
Myocardial infarction	5027 (43.7)	7105 (38.6)	0.104	0.009
Congestive heart failure	986 (8.6)	1082 (5.9)	0.104	0.006
CCS ≥3	4026 (35.0)	6806 (37.0)	0.041	0.001
NYHA ≥3	1630 (14.2)	2578 (14.0)	0.005	0.001
Cardiogenic shock	42 (0.4)	36 (0.2)	0.032	0.005
Resuscitation	43 (0.4)	27 (0.2)	0.045	0.009
Arrhythmia	791 (6.9)	980 (5.3)	0.065	0.001
Left main disease	3356 (29.2)	4886 (26.6)	0.059	0
Number of diseased territories, mean ± SD	2.7 ± 0.5	2.7 ± 0.5	0.001	0.006
Single-vessel disease	183 (1.6)	467 (2.5)	0.067	0.005
Double-vessel disease	3073 (26.7)	4731 (25.7)	0.023	0.003
Triple-vessel disease	8163 (71.0)	13134 (71.4)	0.008	0.005
Perioperative medications				
Inotropes	219 (1.9)	226 (1.2)	0.055	0.005
Nitroglycerin	565 (4.9)	975 (5.3)	0.017	0.006
Anticoagulants	2523 (22.0)	3246 (17.6)	0.108	0.005
Steroids	160 (1.4)	235 (1.3)	0.010	0.001
LVEF measurement method				
Angiogram	4108 (35.8)	8970 (48.8)	0.266	0.004
Radionuclide	156 (1.4)	52 (0.3)	0.119	0.003
Echocardiogram	7076 (61.6)	9218 (50.1)	0.233	0.003
MRI	4 (0.0)	3 (0.0)	0.012	0
Elective	7677 (66.8)	12254 (68.2)	0.031	0.006
Urgent	3574 (31.1)	5506 (29.9)	0.026	0.003
Number of grafts, mean ± SD	3.1 ± 0.9	3.4 ± 1.0	0.368	0.022
On-pump surgery	10888 (94.7)	17113 (93.0)	0.072	0.014

This is part of the primary analyses, where stabilization was applied during weighting. MAG, multiple arterial grafting; SAG, single arterial grafting; LVEF, left ventricular ejection fraction; CCS, Canadian Cardiovascular Society classification; New York Heart Association classification; MRI, magnetic resonance imaging.

© 2025 Ren J et al. *JAMA Network Open.*

eTable 3. Patient Demographics of MAG vs SAG Cohorts With LVEF 46-60%

**Characteristics**<br>Patients, No. (%)	**SAG**	**MAG**	**Unweighted SMD**	**Weighted SMD**
Total sample size	8001	10979	-	-
Age, mean ± SD	67.0 ± 10.2	65.0 ± 10.3	0.200	0.004
Male	6482 (81.0)	9110 (83.0)	0.051	0.003
Body mass index	29.2 ± 9.3	29.0 ± 5.8	0.026	0.006
Smoking history	5288 (66.1)	7222 (65.8)	0.007	0.007
Diabetes	3149 (39.4)	3785 (34.5)	0.101	0.001
Hypercholesterolemia	6538 (81.7)	8895 (81.0)	0.018	0.004
Creatinine, mean ± SD, μmol/L	107.3 ± 99.8	96.1 ± 63.8	0.134	0.004
Dialysis	222 (2.8)	88 (0.8)	0.149	0.002
Hypertension	6581 (82.3)	8566 (78.0)	0.106	0.004
Cerebrovascular event	863 (10.8)	991 (9.0)	0.059	0.001
Peripheral vascular disease	946 (11.8)	1126 (10.3)	0.050	0.003
Respiratory disease	1054 (13.2)	1221 (11.1)	0.063	0.003
Myocardial infarction	4770 (59.6)	6332 (57.7)	0.040	0.003
Congestive heart failure	860 (10.8)	928 (8.5)	0.078	0.002
CCS ≥3	3191 (39.9)	4592 (41.8)	0.040	0.007
NYHA ≥3	1323 (16.5)	1722 (15.7)	0.023	0.002
Cardiogenic shock	74 (0.9)	55 (0.5)	0.050	0.002
Resuscitation	46 (0.6)	36 (0.3)	0.037	0.004
Arrhythmia	767 (9.6)	865 (7.9)	0.061	0.003
Left main disease	2266 (28.3)	2656 (24.2)	0.094	0.004
Number of diseased territories, mean ± SD	2.7 ± 0.5	2.7 ± 0.5	0.007	0.005
Single-vessel disease	97 (1.2)	209 (1.9)	0.056	0.001
Double-vessel disease	1803 (22.5)	2407 (21.9)	0.015	0.004
Triple-vessel disease	6062 (75.8)	8325 (75.8)	0.001	0.005
Perioperative medications				
Inotropes	123 (1.5)	685 (6.2)	0.032	0.003
Nitroglycerin	430 (5.4)	128 (1.2)	0.037	0.001
Anticoagulants	1913 (23.9)	2458 (22.4)	0.036	0.002
Steroids	128 (1.6)	136 (1.2)	0.031	0.004
LVEF measurement method				
Angiogram	2488 (31.1)	4636 (42.2)	0.233	0.004
Radionuclide	81 (1.0)	85 (0.8)	0.025	0
Echocardiogram	5399 (67.5)	6174 (56.2)	0.233	0.007
MRI	11 (0.1)	2 (0.0)	0.043	0.002
Elective	4701 (58.8)	6690 (60.9)	0.045	0.001
Urgent	3037 (38.0)	4020 (36.6)	0.028	0.003
Number of grafts, mean ± SD	3.1 ± 0.9	3.4 ± 1.0	0.309	0.023
On-pump surgery	7638 (95.5)	9933 (90.5)	0.196	0.009

This is part of the primary analyses, where stabilization was applied during weighting. MAG, multiple arterial grafting; SAG, single arterial grafting; LVEF, left ventricular ejection fraction; CCS, Canadian Cardiovascular Society classification; New York Heart Association classification; MRI, magnetic resonance imaging.

© 2025 Ren J et al. *JAMA Network Open.*

eTable 4. Patient Demographics of MAG vs SAG Cohorts With LVEF 30-45%

**Characteristics**<br>Patients, No. (%)	**SAG**	**MAG**	**Unweighted SMD**	**Weighted SMD**
Total sample size	3946	4694	-	-
Age, mean ± SD	66.6 ± 10.6	65.3 ± 10.6	0.123	0.001
Male	3211 (81.4)	3974 (84.7)	0.088	0.008
Body mass index	29.0 ± 9.6	28.9 ± 5.7	0.012	0.003
Smoking history	2719 (68.9)	3305 (70.4)	0.033	0.002
Diabetes	1803 (45.7)	1915 (40.8)	0.099	0.002
Hypercholesterolemia	3145 (79.7)	3662 (78.0)	0.041	0.006
Creatinine, mean ± SD, µmol/L	118.7 ± 121.3	102.4 ± 73.8	0.162	0.028
Dialysis	152 (3.9)	52 (1.1)	0.177	0.026
Hypertension	6444 (82.2)	3652 (77.8)	0.109	0.007
Cerebrovascular event	516 (13.1)	547 (11.7)	0.043	0.012
Peripheral vascular disease	586 (14.9)	675 (14.4)	0.013	0.011
Respiratory disease	644 (16.3)	689 (14.7)	0.045	0.003
Myocardial infarction	3039 (77.0)	3531 (75.2)	0.042	0.002
Congestive heart failure	1170 (29.7)	1128 (24.0)	0.127	0.010
CCS ≥3	1690 (42.8)	2104 (44.8)	0.040	0.006
NYHA ≥3	1072 (27.2)	1190 (25.4)	0.041	0.013
Cardiogenic shock	156 (3.9)	100 (2.1)	0.106	0.038
Resuscitation	71 (1.8)	47 (1.0)	0.068	0.014
Arrhythmia	586 (14.9)	602 (12.8)	0.059	0.004
Left main disease	1125 (28.5)	1184 (25.2)	0.074	0.008
Number of diseased territories, mean ± SD	2.8 ± 0.5	2.8 ± 0.5	0.014	0.005
Single-vessel disease	34 (0.9)	66 (1.4)	0.051	0.006
Double-vessel disease	692 (17.5)	805 (17.2)	0.010	0.001
Triple-vessel disease	3204 (81.2)	3805 (81.1)	0.004	0.003
Perioperative medications				
Inotropes	156 (4.0)	86 (1.8)	0.127	0.033
Nitroglycerin	293 (7.4)	389 (8.3)	0.032	0.019
Anticoagulants	1205 (30.5)	1215 (25.9)	0.104	0.003
Steroids	55 (1.4)	72 (1.5)	0.012	0
LVEF measurement method				
Angiogram	1096 (27.8)	1848 (39.4)	0.247	0.004
Radionuclide	85 (2.2)	80 (1.7)	0.033	0.003
Echocardiogram	2725 (69.1)	2729 (58.1)	0.228	0.005
MRI	24 (0.6)	15 (0.3)	0.043	0.002
Elective	2037 (51.6)	2641 (56.3)	0.093	0.001
Urgent	1659 (42.0)	1817 (38.7)	0.068	0
Number of grafts, mean ± SD	3.2 ± 0.9	3.5 ± 1.0	0.308	0.015
On-pump surgery	3790 (96.1)	4328 (92.2)	0.164	0.026

This is part of the primary analyses, where stabilization was applied during weighting. MAG, multiple arterial grafting; SAG, single arterial grafting; LVEF, left ventricular ejection fraction; CCS, Canadian Cardiovascular Society classification; New York Heart Association classification; MRI, magnetic resonance imaging.

© 2025 Ren J et al. *JAMA Network Open.*

eTable 5. Patient Demographics of MAG vs SAG Cohorts With LVEF <30%

**Characteristics**<br>Patients, No. (%)	**SAG**	**MAG**	**Unweighted SMD**	**Weighted SMD**
Total sample size	1125	1005	-	-
Age, mean ± SD	65.4 ± 10.8	64.7 ± 10.6	0.065	0.004
Male	957 (85.1)	856 (85.2)	0.003	0.013
Body mass index	28.5 ± 13.1	28.4 ± 7.7	0.006	0.004
Smoking history	791 (70.3)	744 (74.0)	0.083	0.008
Diabetes	562 (50.0)	488 (48.6)	0.028	0.001
Hypercholesterolemia	873 (77.6)	777 (77.3)	0.007	0.026
Creatinine, mean ± SD, μmol/L	121.0 ± 107.6	108.0 ± 62.9	0.147	0.029
Dialysis	44 (3.9)	12 (1.2)	0.173	0.007
Hypertension	870 (77.3)	755 (75.1)	0.052	0.003
Cerebrovascular event	142 (12.6)	107 (10.7)	0.062	0.006
Peripheral vascular disease	175 (15.6)	140 (13.9)	0.046	0.007
Respiratory disease	180 (16.0)	156 (15.2)	0.013	0.013
Myocardial infarction	872 (77.5)	796 (79.2)	0.041	0.010
Congestive heart failure	583 (51.8)	523 (52.0)	0.004	0.012
CCS ≥3	481 (42.8)	489 (48.7)	0.119	0.012
NYHA ≥3	534 (47.5)	467 (46.5)	0.020	0.023
Cardiogenic shock	128 (11.4)	63 (6.3)	0.181	0.039
Resuscitation	52 (4.6)	33 (3.3)	0.069	0.035
Arrhythmia	211 (18.8)	204 (20.3)	0.039	0.007
Left main disease	346 (30.7)	293 (29.2)	0.035	0.006
Number of diseased territories, mean ± SD	2.8 ± 0.5	2.8 ± 0.4	0.098	0.002
Single-vessel disease	7 (0.6)	10 (1.0)	0.042	0.014
Double-vessel disease	193 (17.2)	137 (13.6)	0.098	0.016
Triple-vessel disease	917 (81.5)	856 (85.2)	0.098	0.010
Perioperative medications				
Inotropes	144 (12.8)	73 (7.3)	0.185	0.027
Nitroglycerin	114 (10.1)	108 (10.8)	0.020	0.011
Anticoagulants	377 (33.5)	353 (35.1)	0.034	0.007
Steroids	20 (1.8)	23 (2.3)	0.036	0.016
LVEF measurement method				
Angiogram	232 (20.6)	376 (37.4)	0.377	0.005
Radionuclide	66 (5.9)	45 (4.5)	0.063	0.018
Echocardiogram	808 (71.8)	563 (56.0)	0.334	0.006
MRI	16 (1.4)	11 (1.1)	0.029	0.011
Elective	529 (47.0)	492 (48.9)	0.039	0.004
Urgent	458 (40.7)	417 (41.5)	0.016	0.010
Number of grafts, mean ± SD	3.3 ± 1.0	3.7 ± 1.0	0.345	0.042
On-pump surgery	1075 (95.6)	958 (95.3)	0.011	0.010

This is part of the primary analyses, where stabilization was applied during weighting. MAG, multiple arterial grafting; SAG, single arterial grafting; LVEF, left ventricular ejection fraction; CCS, Canadian Cardiovascular Society classification; New York Heart Association classification; MRI, magnetic resonance imaging.

© 2025 Ren J et al. *JAMA Network Open.*

eTable 6. Patient Demographics of MAG-TAR and MAG-SVG Cohorts With LVEF >60%

**Characteristics**<br>Patients, No. (%)	**MAG-TAR**	**MAG-SVG**	**Unweighted SMD**	**Weighted SMD**
Total sample size	9386	9013	-	-
Age, mean ± SD	64.0 ± 10.1	66.4 ± 9.6	0.242	0
Male	7482 (79.7)	7373 (81.8)	0.053	0.001
Body mass index	29.0 ± 6.9	29.0 ± 7.2	0.001	0.007
Smoking history	5825 (62.1)	5444 (60.4)	0.035	0.004
Diabetes	2779 (29.6)	3113 (34.5)	0.106	0.002
Hypercholesterolemia	7700 (82.0)	7336 (81.4)	0.017	0.019
Creatinine, mean ± SD, µmol/L	91.0 ± 52.2	93.5 ± 59.0	0.046	0.009
Dialysis	27 (0.3)	55 (0.6)	0.048	0.006
Hypertension	7191 (76.6)	7142 (79.2)	0.063	0.006
Cerebrovascular event	740 (7.9)	793 (8.8)	0.033	0.015
Peripheral vascular disease	781 (8.3)	767 (8.5)	0.007	0.001
Respiratory disease	905 (9.6)	835 (9.3)	0.013	0.009
Myocardial infarction	3400 (36.2)	3705 (41.1)	0.100	0.005
Congestive heart failure	574 (6.1)	508 (5.6)	0.020	0.003
CCS ≥3	3550 (37.8)	3256 (36.1)	0.035	0.012
NYHA ≥3	1418 (15.1)	1160 (12.9)	0.065	0.003
Cardiogenic shock	17 (0.2)	19 (0.2)	0.007	0.002
Resuscitation	8 (0.1)	19 (0.2)	0.033	0.011
Arrhythmia	478 (5.1)	502 (5.6)	0.021	0.024
Left main disease	2747 (25.9)	2459 (27.3)	0.032	0.007
Number of diseased territories, mean ± SD	2.5 ± 0.6	2.9 ± 0.4	0.662	0.037
Single-vessel disease	447 (4.8)	20 (0.2)	0.294	0.036
Double-vessel disease	3562 (38.0)	1169 (13.0)	0.599	0.021
Triple-vessel disease	5348 (57.0)	7786 (86.4)	0.691	0.032
Perioperative medications				
Inotropes	50 (0.5)	176 (2.0)	0.129	0.021
Nitroglycerin	446 (4.8)	529 (5.9)	0.050	0.003
Anticoagulants	1470 (15.6)	176 (19.7)	0.106	0.007
Steroids	107 (1.1)	128 (1.4)	0.025	0.002
LVEF measurement method				
Angiogram	4497 (47.9)	4473 (49.6)	0.034	0.005
Radionuclide	27 (0.3)	25 (0.3)	0.002	0.012
Echocardiogram	4789 (51.0)	4429 (49.1)	0.038	0.001
MRI	2 (0.02)	1 (0.01)	0.008	0.003
Elective	6434 (68.6)	6120 (67.9)	0.014	0.008
Urgent	2776 (29.6)	2730 (30.3)	0.016	0.004
Number of grafts, mean ± SD	3.0 ± 0.9	3.8 ± 0.9	0.880	0.016
On-pump surgery	8408 (89.6)	8705 (96.6)	0.279	0.028

MAG, multiple arterial grafting; SAG, single arterial grafting; LVEF, left ventricular ejection fraction; CCS, Canadian Cardiovascular Society classification; New York Heart Association classification; MRI, magnetic resonance imaging.

© 2025 Ren J et al. *JAMA Network Open.*

eTable 7. Patient Demographics of MAG-TAR and MAG-SVG Cohorts With LVEF 46-60%

**Characteristics**<br>Patients, No. (%)	**MAG-TAR**	**MAG-SVG**	**Unweighted SMD**	**Weighted SMD**
Total sample size	5278	5701	-	-
Age, mean ± SD	64.2 ± 10.5	65.7 ± 10.0	0.152	0.010
Male	4282 (81.1)	4828 (84.7)	0.095	0.010
Body mass index	29.0 ± 5.2	29.1 ± 6.3	0.010	0
Smoking history	3502 (66.4)	3720 (65.3)	0.023	0.003
Diabetes	1743 (33.0)	2042 (35.8)	0.059	0.012
Hypercholesterolemia	4383 (81.5)	4592 (80.6)	0.025	0.009
Creatinine, mean ± SD, µmol/L	95.4 ± 62.9	96.7 ± 64.6	0.021	0.032
Dialysis	29 (0.6)	59 (1.0)	0.055	0.035
Hypertension	4087 (77.4)	4479 (78.6)	0.027	0.002
Cerebrovascular event	489 (9.3)	502 (8.8)	0.016	0.031
Peripheral vascular disease	593 (11.2)	533 (9.4)	0.062	0.019
Respiratory disease	603 (11.4)	618 (10.8)	0.019	0.019
Myocardial infarction	2962 (56.1)	3370 (59.1)	0.061	0
Congestive heart failure	473 (9.0)	455 (8.0)	0.035	0.015
CCS ≥3	2334 (44.2)	2258 (39.6)	0.094	0.004
NYHA ≥3	879 (16.7)	843 (14.8)	0.051	0.001
Cardiogenic shock	16 (0.3)	39 (0.7)	0.054	0.006
Resuscitation	14 (0.3)	22 (0.4)	0.021	0.012
Arrhythmia	411 (7.8)	454 (8.0)	0.007	0.019
Left main disease	1228 (23.3)	1428 (25.1)	0.042	0.016
Number of diseased territories, mean ± SD	2.6 ± 0.6	2.9 ± 0.4	0.589	0.046
Single-vessel disease	197 (3.7)	12 (0.2)	0.256	0.038
Double-vessel disease	1759 (33.3)	648 (11.4)	0.547	0.024
Triple-vessel disease	3306 (62.6)	5019 (88.0)	0.617	0.038
Perioperative medications				
Inotropes	30 (0.6)	98 (1.7)	0.108	0.005
Nitroglycerin	314 (6.0)	371 (6.5)	0.023	0.010
Anticoagulants	1081 (20.5)	1377 (24.2)	0.088	0.031
Steroids	59 (1.1)	77 (1.4)	0.021	0.004
LVEF measurement method				
Angiogram	2139 (40.5)	2497 (43.8)	0.066	0.019
Radionuclide	45 (0.9)	40 (0.7)	0.017	0.008
Echocardiogram	3047 (57.7)	3127 (54.8)	0.058	0.020
MRI	0 (0.0)	2 (0.0)	0.027	0.015
Elective	3259 (61.8)	3431 (60.2)	0.032	0.022
Urgent	1898 (36.0)	2122 (37.2)	0.026	0.020
Number of grafts, mean ± SD	3.0 ± 0.9	3.8 ± 0.9	0.879	0.084
On-pump surgery	4424 (83.8)	5509 (96.6)	0.442	0.053

MAG, multiple arterial grafting; SAG, single arterial grafting; LVEF, left ventricular ejection fraction; CCS, Canadian Cardiovascular Society classification; New York Heart Association classification; MRI, magnetic resonance imaging.

© 2025 Ren J et al. *JAMA Network Open.*

eTable 8. Patient Demographics of MAG-TAR and MAG-SVG Cohorts With LVEF 30-45%

**Characteristics**<br>Patients, No. (%)	**MAG-TAR**	**MAG-SVG**	**Unweighted SMD**	**Weighted SMD**
Total sample size	1980	2714	-	-
Age, mean ± SD	64.8 ± 10.8	65.7 ± 10.3	0.078	0.013
Male	1622 (81.9)	2352 (86.7)	0.131	0.012
Body mass index	29.0 ± 6.2	28.9 ± 5.3	0.016	0.010
Smoking history	1408 (71.1)	1897 (69.9)	0.027	0.016
Diabetes	757 (38.2)	1158 (42.7)	0.091	0.001
Hypercholesterolemia	1554 (78.5)	2108 (77.7)	0.020	0
Creatinine, mean ± SD, µmol/L	99.6 ± 63.6	104.5 ± 80.4	0.066	0.008
Dialysis	23 (1.2)	29 (1.1)	0.009	0.002
Hypertension	1538 (77.7)	2114 (77.9)	0.005	0.010
Cerebrovascular event	219 (11.1)	328 (12.1)	0.032	0.001
Peripheral vascular disease	286 (14.4)	389 (14.3)	0.003	0.003
Respiratory disease	284 (14.3)	405 (14.9)	0.016	0.005
Myocardial infarction	1477 (74.6)	2054 (75.7)	0.025	0.010
Congestive heart failure	463 (23.4)	665 (24.5)	0.026	0
CCS ≥3	916 (46.3)	1188 (43.8)	0.050	0.005
NYHA ≥3	488 (24.7)	702 (25.9)	0.028	0.002
Cardiogenic shock	29 (1.5)	71 (2.6)	0.082	0.001
Resuscitation	10 (0.5)	37 (1.4)	0.089	0.001
Arrhythmia	248 (12.5)	354 (13.0)	0.016	0.005
Left main disease	470 (23.7)	714 (26.3)	0.059	0
Number of diseased territories, mean ± SD	2.6 ± 0.6	2.9 ± 0.3	0.534	0.003
Single-vessel disease	58 (2.9)	8 (0.3)	0.210	0.001
Double-vessel disease	555 (28.0)	250 (9.2)	0.498	0.008
Triple-vessel disease	1356 (68.5)	2449 (90.2)	0.558	0.006
Perioperative medications				
Inotropes	24 (2.1)	62 (2.3)	0.082	0.006
Nitroglycerin	166 (8.4)	223 (8.2)	0.006	0.010
Anticoagulants	477 (24.1)	738 (27.2)	0.071	0.010
Steroids	28 (1.4)	44 (1.6)	0.017	0.001
LVEF measurement method				
Angiogram	757 (38.2)	1091 (40.2)	0.040	0.002
Radionuclide	33 (1.7)	47 (1.7)	0.005	0.007
Echocardiogram	1177 (59.4)	1552 (57.2)	0.046	0.005
MRI	2 (0.1)	13 (0.5)	0.070	0.011
Elective	1181 (59.7)	1460 (53.8)	0.118	0.011
Urgent	716 (36.2)	1101 (40.6)	0.091	0.010
Number of grafts, mean ± SD	3.1 ± 0.9	3.9 ± 0.9	0.896	0.004
On-pump surgery	1707 (86.2)	2621 (96.6)	0.376	0.016

MAG, multiple arterial grafting; SAG, single arterial grafting; LVEF, left ventricular ejection fraction; CCS, Canadian Cardiovascular Society classification; New York Heart Association classification; MRI, magnetic resonance imaging.

© 2025 Ren J et al. *JAMA Network Open.*

eTable 9. Patient Demographics of MAG-TAR and MAG-SVG Cohorts With LVEF <30%

**Characteristics**<br>Patients, No. (%)	**MAG-TAR**	**MAG-SVG**	**Unweighted SMD**	**Weighted SMD**
Total sample size	394	611	-	-
Age, mean ± SD	64.3 ± 11.5	65.0 ± 9.9	0.067	0.001
Male	333 (84.5)	523 (85.6)	0.031	0.006
Body mass index	28.7 ± 10.2	28.3 ± 5.6	0.056	0.007
Smoking history	302 (76.7)	442 (72.3)	0.099	0.008
Diabetes	180 (45.7)	308 (50.4)	0.095	0.001
Hypercholesterolemia	301 (76.4)	476 (77.9)	0.036	0
Creatinine, mean ± SD, μmol/L	102.1 ± 55.2	111.8 ± 67.2	0.183	0.030
Dialysis	3 (0.8)	9 (1.5)	0.073	0.020
Hypertension	281 (71.3)	474 (77.6)	0.142	0.052
Cerebrovascular event	43 (10.9)	64 (10.5)	0.014	0.009
Peripheral vascular disease	57 (14.5)	83 (13.6)	0.024	0.011
Respiratory disease	71 (18.0)	85 (13.9)	0.110	0.011
Myocardial infarction	306 (77.7)	490 (80.2)	0.062	0.015
Congestive heart failure	203 (51.5)	320 (52.4)	0.017	0.003
CCS ≥3	202 (51.3)	287 (47.0)	0.086	0.008
NYHA ≥3	192 (48.7)	275 (45.0)	0.075	0.006
Cardiogenic shock	20 (5.1)	43 (7.0)	0.084	0.005
Resuscitation	8 (2.0)	25 (4.1)	0.131	0.001
Arrhythmia	82 (20.8)	122 (20.0)	0.021	0.001
Left main disease	103 (26.1)	190 (31.1)	0.112	0.011
Number of diseased territories, mean ± SD	2.7 ±0.5	2.9 ± 0.3	0.569	0.002
Single-vessel disease	8 (2.0)	2 (0.3)	0.242	0.021
Double-vessel disease	95 (24.1)	42 (6.9)	0.516	0.007
Triple-vessel disease	290 (73.6)	566 (92.6)	0.560	0.002
Perioperative medications				
Inotropes	21 (5.3)	52 (8.5)	0.130	0.005
Nitroglycerin	42 (10.7)	66 (10.8)	0.132	0.001
Anticoagulants	123 (31.2)	230 (37.6)	0.136	0.006
Steroids	6 (1.5)	17 (2.8)	0.101	0.013
LVEF measurement method				
Angiogram	137 (34.8)	239 (39.1)	0.089	0.013
Radionuclide	21 (5.3)	24 (3.9)	0.065	0.002
Echocardiogram	228 (57.9)	335 (54.8)	0.061	0.013
MRI	4 (1.0)	7 (1.2)	0.017	0.003
Elective	195 (49.5)	297 (48.6)	0.018	0.014
Urgent	169 (42.9)	248 (40.6)	0.047	0.019
Number of grafts, mean ± SD	3.2 ± 1.0	4.0 ± 0.9	0.904	0.002
On-pump surgery	354 (89.9)	604 (98.9)	0.605	0.008

MAG, multiple arterial grafting; SAG, single arterial grafting; LVEF, left ventricular ejection fraction; CCS, Canadian Cardiovascular Society classification; New York Heart Association classification; MRI, magnetic resonance imaging.

© 2025 Ren J et al. *JAMA Network Open.*

eTable 10. Patient Demographics of MAG and SVG Cohorts in Sensitivity Analysis Without Stabilization During Inverse Probability Weighting (LVEF >60%)

**Characteristics Patients, No. (%)**	**SAG**	**MAG**	**Unweighted SMD**	**Weighted SMD**
Total sample size	11492	18399	-	-
Age, mean ± SD	67.1 ± 9.8	65.1 ± 10.0	0.196	0.006
Male	8876 (77.2)	14855 (80.7)	0.086	0.002
Body mass index	29.1 ± 8.6	29.0 ± 7.0	0.007	0.006
Smoking history	7254 (63.1)	11267 (61.2)	0.039	0.008
Diabetes	4267 (37.1)	5892 (32.0)	0.108	0.004
Hypercholesterolemia	9300 (81.7)	15036 (81.7)	0	0.010
Creatinine, mean ± SD, µmol/L	99.1 ± 80.3	92.2 ± 55.7	0.099	0.012
Dialysis	179 (1.6)	82 (0.5)	0.112	0.002
Hypertension	9459 (82.1)	14333 (77.9)	0.111	0
Cerebrovascular event	1143 (9.95)	1533 (8.3)	0.056	0.001
Peripheral vascular disease	1089 (9.5)	1548 (8.4)	0.037	0.004
Respiratory disease	1323 (11.5)	1740 (9.5)	0.067	0
Myocardial infarction	5027 (43.7)	7105 (38.6)	0.104	0.009
Congestive heart failure	986 (8.6)	1082 (5.9)	0.104	0.006
CCS ≥3	4026 (35.0)	6806 (37.0)	0.041	0.001
NYHA ≥3	1630 (14.2)	2578 (14.0)	0.005	0.001
Cardiogenic shock	42 (0.4)	36 (0.2)	0.032	0.005
Resuscitation	43 (0.4)	27 (0.2)	0.045	0.009
Arrhythmia	791 (6.9)	980 (5.3)	0.065	0.001
Left main disease	3356 (29.2)	4886 (26.6)	0.059	0
Number of diseased territories, mean ± SD	2.7 ± 0.5	2.7 ± 0.5	0.001	0.006
Single-vessel disease	183 (1.6)	467 (2.5)	0.067	0.005
Double-vessel disease	3073 (26.7)	4731 (25.7)	0.023	0.003
Triple-vessel disease	8163 (71.0)	13134 (71.4)	0.008	0.005
Perioperative medications				
Inotropes	219 (1.9)	226 (1.2)	0.055	0.005
Nitroglycerin	565 (4.9)	975 (5.3)	0.017	0.006
Anticoagulants	2523 (22.0)	3246 (17.6)	0.108	0.005
Steroids	160 (1.4)	235 (1.3)	0.010	0.001
LVEF measurement method				
Angiogram	4108 (35.8)	8970 (48.8)	0.266	0.004
Radionuclide	156 (1.4)	52 (0.3)	0.119	0.003
Echocardiogram	7076 (61.6)	9218 (50.1)	0.233	0.003
MRI	4 (0.0)	3 (0.0)	0.012	0
Elective	7677 (66.8)	12254 (68.2)	0.031	0.006
Urgent	3574 (31.1)	5506 (29.9)	0.026	0.003
Number of grafts, mean ± SD	3.1 ± 0.9	3.4 ± 1.0	0.368	0.022
On-pump surgery	10888 (94.7)	17113 (93.0)	0.072	0.014

MAG, multiple arterial grafting; SAG, single arterial grafting; LVEF, left ventricular ejection fraction; CCS, Canadian Cardiovascular Society classification; New York Heart Association classification; MRI, magnetic resonance imaging.

eTable 11. Patient Demographics of MAG and SVG Cohorts in Sensitivity Analysis Without Stabilization During Inverse Probability Weighting (LVEF 46-60%)

**Characteristics**<br>Patients, No. (%)	**SAG**	**MAG**	**Unweighted SMD**	**Weighted SMD**
Total sample size	8001	10979	-	-
Age, mean ± SD	67.0 ± 10.2	65.0 ± 10.3	0.200	0.004
Male	6482 (81.0)	9110 (83.0)	0.051	0.003
Body mass index	29.2 ± 9.3	29.0 ± 5.8	0.026	0.006
Smoking history	5288 (66.1)	7222 (65.8)	0.007	0.007
Diabetes	3149 (39.4)	3785 (34.5)	0.101	0.001
Hypercholesterolemia	6538 (81.7)	8895 (81.0)	0.018	0.004
Creatinine, mean ± SD, µmol/L	107.3 ± 99.8	96.1 ± 63.8	0.134	0.004
Dialysis	222 (2.8)	88 (0.8)	0.149	0.002
Hypertension	6581 (82.3)	8566 (78.0)	0.106	0.004
Cerebrovascular event	863 (10.8)	991 (9.0)	0.059	0.001
Peripheral vascular disease	946 (11.8)	1126 (10.3)	0.050	0.003
Respiratory disease	1054 (13.2)	1221 (11.1)	0.063	0.003
Myocardial infarction	4770 (59.6)	6332 (57.7)	0.040	0.003
Congestive heart failure	860 (10.8)	928 (8.5)	0.078	0.002
CCS ≥3	3191 (39.9)	4592 (41.8)	0.040	0.007
NYHA ≥3	1323 (16.5)	1722 (15.7)	0.023	0.002
Cardiogenic shock	74 (0.9)	55 (0.5)	0.050	0.002
Resuscitation	46 (0.6)	36 (0.3)	0.037	0.004
Arrhythmia	767 (9.6)	865 (7.9)	0.061	0.003
Left main disease	2266 (28.3)	2656 (24.2)	0.094	0.004
Number of diseased territories, mean ± SD	2.7 ± 0.5	2.7 ± 0.5	0.007	0.005
Single-vessel disease	97 (1.2)	209 (1.9)	0.056	0.001
Double-vessel disease	1803 (22.5)	2407 (21.9)	0.015	0.004
Triple-vessel disease	6062 (75.8)	8325 (75.8)	0.001	0.005
Perioperative medications				
Inotropes	123 (1.5)	685 (6.2)	0.032	0.003
Nitroglycerin	430 (5.4)	128 (1.2)	0.037	0.001
Anticoagulants	1913 (23.9)	2458 (22.4)	0.036	0.002
Steroids	128 (1.6)	136 (1.2)	0.031	0.004
LVEF measurement method				
Angiogram	2488 (31.1)	4636 (42.2)	0.233	0.004
Radionuclide	81 (1.0)	85 (0.8)	0.025	0
Echocardiogram	5399 (67.5)	6174 (56.2)	0.233	0.007
MRI	11 (0.1)	2 (0.0)	0.043	0.002
Elective	4701 (58.8)	6690 (60.9)	0.045	0.001
Urgent	3037 (38.0)	4020 (36.6)	0.028	0.003
Number of grafts, mean ± SD	3.1 ± 0.9	3.4 ± 1.0	0.309	0.023
On-pump surgery	7638 (95.5)	9933 (90.5)	0.196	0.009

MAG, multiple arterial grafting; SAG, single arterial grafting; LVEF, left ventricular ejection fraction; CCS, Canadian Cardiovascular Society classification; New York Heart Association classification; MRI, magnetic resonance imaging.

© 2025 Ren J et al. *JAMA Network Open.*

eTable 12. Patient Demographics of MAG and SVG Cohorts in Sensitivity Analysis Without Stabilization During Inverse Probability Weighting (LVEF 30-45%)

**Characteristics**<br>Patients, No. (%)	**SAG**	**MAG**	**Unweighted SMD**	**Weighted SMD**
Total sample size	3946	4694	-	-
Age, mean ± SD	66.6 ± 10.6	65.3 ± 10.6	0.123	0.001
Male	3211 (81.4)	3974 (84.7)	0.088	0.008
Body mass index	29.0 ± 9.6	28.9 ± 5.7	0.012	0.003
Smoking history	2719 (68.9)	3305 (70.4)	0.033	0.002
Diabetes	1803 (45.7)	1915 (40.8)	0.099	0.002
Hypercholesterolemia	3145 (79.7)	3662 (78.0)	0.041	0.006
Creatinine, mean ± SD, µmol/L	118.7 ± 121.3	102.4 ± 73.8	0.162	0.028
Dialysis	152 (3.9)	52 (1.1)	0.177	0.026
Hypertension	6444 (82.2)	3652 (77.8)	0.109	0.007
Cerebrovascular event	516 (13.1)	547 (11.7)	0.043	0.012
Peripheral vascular disease	586 (14.9)	675 (14.4)	0.013	0.011
Respiratory disease	644 (16.3)	689 (14.7)	0.045	0.003
Myocardial infarction	3039 (77.0)	3531 (75.2)	0.042	0.002
Congestive heart failure	1170 (29.7)	1128 (24.0)	0.127	0.010
CCS ≥3	1690 (42.8)	2104 (44.8)	0.040	0.006
NYHA ≥3	1072 (27.2)	1190 (25.4)	0.041	0.013
Cardiogenic shock	156 (3.9)	100 (2.1)	0.106	0.038
Resuscitation	71 (1.8)	47 (1.0)	0.068	0.014
Arrhythmia	586 (14.9)	602 (12.8)	0.059	0.004
Left main disease	1125 (28.5)	1184 (25.2)	0.074	0.008
Number of diseased territories, mean ± SD	2.8 ± 0.5	2.8 ± 0.5	0.014	0.005
Single-vessel disease	34 (0.9)	66 (1.4)	0.051	0.006
Double-vessel disease	692 (17.5)	805 (17.2)	0.010	0.001
Triple-vessel disease	3204 (81.2)	3805 (81.1)	0.004	0.003
Perioperative medications				
Inotropes	156 (4.0)	86 (1.8)	0.127	0.033
Nitroglycerin	293 (7.4)	389 (8.3)	0.032	0.019
Anticoagulants	1205 (30.5)	1215 (25.9)	0.104	0.003
Steroids	55 (1.4)	72 (1.5)	0.012	0
LVEF measurement method				
Angiogram	1096 (27.8)	1848 (39.4)	0.247	0.004
Radionuclide	85 (2.2)	80 (1.7)	0.033	0.003
Echocardiogram	2725 (69.1)	2729 (58.1)	0.228	0.005
MRI	24 (0.6)	15 (0.3)	0.043	0.002
Elective	2037 (51.6)	2641 (56.3)	0.093	0.001
Urgent	1659 (42.0)	1817 (38.7)	0.068	0
Number of grafts, mean ± SD	3.2 ± 0.9	3.5 ± 1.0	0.308	0.015
On-pump surgery	3790 (96.1)	4328 (92.2)	0.164	0.026

MAG, multiple arterial grafting; SAG, single arterial grafting; LVEF, left ventricular ejection fraction; CCS, Canadian Cardiovascular Society classification; New York Heart Association classification; MRI, magnetic resonance imaging.

© 2025 Ren J et al. *JAMA Network Open.*

eTable 13. Patient Demographics of MAG and SVG Cohorts in Sensitivity Analysis Without Stabilization During Inverse Probability Weighting (LVEF <30%)

**Characteristics**<br>Patients, No. (%)	**SAG**	**MAG**	**Unweighted SMD**	**Weighted SMD**
Total sample size	1125	1005	-	-
Age, mean ± SD	65.4 ± 10.8	64.7 ± 10.6	0.065	0.004
Male	957 (85.1)	856 (85.2)	0.003	0.013
Body mass index	28.5 ± 13.1	28.4 ± 7.7	0.006	0.004
Smoking history	791 (70.3)	744 (74.0)	0.083	0.008
Diabetes	562 (50.0)	488 (48.6)	0.028	0.001
Hypercholesterolemia	873 (77.6)	777 (77.3)	0.007	0.026
Creatinine, mean ± SD, µmol/L	121.0 ± 107.6	108.0 ± 62.9	0.147	0.029
Dialysis	44 (3.9)	12 (1.2)	0.173	0.007
Hypertension	870 (77.3)	755 (75.1)	0.052	0.003
Cerebrovascular event	142 (12.6)	107 (10.7)	0.062	0.006
Peripheral vascular disease	175 (15.6)	140 (13.9)	0.046	0.007
Respiratory disease	180 (16.0)	156 (15.2)	0.013	0.013
Myocardial infarction	872 (77.5)	796 (79.2)	0.041	0.010
Congestive heart failure	583 (51.8)	523 (52.0)	0.004	0.012
CCS ≥3	481 (42.8)	489 (48.7)	0.119	0.012
NYHA ≥3	534 (47.5)	467 (46.5)	0.020	0.023
Cardiogenic shock	128 (11.4)	63 (6.3)	0.181	0.039
Resuscitation	52 (4.6)	33 (3.3)	0.069	0.035
Arrhythmia	211 (18.8)	204 (20.3)	0.039	0.007
Left main disease	346 (30.7)	293 (29.2)	0.035	0.006
Number of diseased territories, mean ± SD	2.8 ± 0.5	2.8 ± 0.4	0.098	0.002
Single-vessel disease	7 (0.6)	10 (1.0)	0.042	0.014
Double-vessel disease	193 (17.2)	137 (13.6)	0.098	0.016
Triple-vessel disease	917 (81.5)	856 (85.2)	0.098	0.010
Perioperative medications				
Inotropes	144 (12.8)	73 (7.3)	0.185	0.027
Nitroglycerin	114 (10.1)	108 (10.8)	0.020	0.011
Anticoagulants	377 (33.5)	353 (35.1)	0.034	0.007
Steroids	20 (1.8)	23 (2.3)	0.036	0.016
LVEF measurement method				
Angiogram	232 (20.6)	376 (37.4)	0.377	0.005
Radionuclide	66 (5.9)	45 (4.5)	0.063	0.018
Echocardiogram	808 (71.8)	563 (56.0)	0.334	0.006
MRI	16 (1.4)	11 (1.1)	0.029	0.011
Elective	529 (47.0)	492 (48.9)	0.039	0.004
Urgent	458 (40.7)	417 (41.5)	0.016	0.010
Number of grafts, mean ± SD	3.3 ± 1.0	3.7 ± 1.0	0.345	0.042
On-pump surgery	1075 (95.6)	958 (95.3)	0.011	0.010

MAG, multiple arterial grafting; SAG, single arterial grafting; LVEF, left ventricular ejection fraction; CCS, Canadian Cardiovascular Society classification; New York Heart Association classification; MRI, magnetic resonance imaging.

© 2025 Ren J et al. *JAMA Network Open.*

eTable 14. Patient Demographics of MAG and SVG Cohorts Before and After Propensity Score Matching (LVEF >60%)

	Before PSM			After PSM		
**Characteristics Patients, No. (%)**	**SAG**	**MAG**	**SMD**	**SAG**	**MAG**	**SMD**
Total sample size	11492	18399	-	10891	10891	-
Age, mean ± SD	67.1 ± 9.8	65.1 ± 10.0	0.196	66.9 ± 9.8	66.3 ± 9.8	0.054
Male	8876 (77.2)	14855 (80.7)	0.086	8467 (77.7)	8564 (78.6)	0.023
Body mass index	29.1 ± 8.6	29.0 ± 7.0	0.007	29.1 ± 8.8	29.1 ± 5.6	0
Smoking history	7254 (63.1)	11267 (61.2)	0.039	6848 (62.9)	6700 (61.5)	0.029
Diabetes	4267 (37.1)	5892 (32.0)	0.108	3946 (36.2)	3866 (35.5)	0.016
Hypercholesterolemia	9300 (81.7)	15036 (81.7)	0	8933 (81.5)	8990 (81.8)	0.004
Creatinine, mean ± SD, μmol/L	99.1 ± 80.3	92.2 ± 55.7	0.099	96.5 ± 40.2	95.4 ± 62.5	0.028
Dialysis	179 (1.6)	82 (0.5)	0.112	92 (0.8)	73 (0.7)	0.026
Hypertension	9459 (82.1)	14333 (77.9)	0.111	9087 (81.7)	9083 (82.3)	0.023
Cerebrovascular event	1143 (9.95)	1533 (8.3)	0.056	1037 (9.5)	973 (8.9)	0.021
Peripheral vascular disease	1089 (9.5)	1548 (8.4)	0.037	1016 (9.3)	972 (8.9)	0.015
Respiratory disease	1323 (11.5)	1740 (9.5)	0.067	1120 (11.1)	1156 (10.6)	0.017
Myocardial infarction	5027 (43.7)	7105 (38.6)	0.104	4651 (42.7)	4483 (41.2)	0.032
Congestive heart failure	986 (8.6)	1082 (5.9)	0.104	827 (7.6)	740 (6.8)	0.034
CCS ≥3	4026 (35.0)	6806 (37.0)	0.041	3814 (35.0)	3905 (35.9)	0.017
NYHA ≥3	1630 (14.2)	2578 (14.0)	0.005	1514 (13.9)	1549 (14.2)	0.009
Cardiogenic shock	42 (0.4)	36 (0.2)	0.032	32 (0.3)	26 (0.2)	0.013
Resuscitation	43 (0.4)	27 (0.2)	0.045	30 (0.3)	22 (0.2)	0.019
Arrhythmia	791 (6.9)	980 (5.3)	0.065	711 (6.5)	669 (6.1)	0.017
Left main disease	3356 (29.2)	4886 (26.6)	0.059	3158 (28.9)	3045 (27.9)	0.023
Number of diseased territories, mean ± SD	2.7 ± 0.5	2.7 ± 0.5	0.001	2.5 ± 0.5	2.5 ± 0.5	0.010
Single-vessel disease	183 (1.6)	467 (2.5)	0.067	182 (1.7)	217 (2.0)	0.020
Double-vessel disease	3073 (26.7)	4731 (25.7)	0.023	2947 (27.1)	2964 (27.2)	0.004
Triple-vessel disease	8163 (71.0)	13134 (71.4)	0.008	7699 (70.7)	7657 (70.3)	0.009
Perioperative medications						
Inotropes	219 (1.9)	226 (1.2)	0.055	205 (1.9)	181 (1.7)	0.002
Nitroglycerin	565 (4.9)	975 (5.3)	0.017	543 (5.0)	557 (5.1)	0.006
Anticoagulants	2523 (22.0)	3246 (17.6)	0.108	2308 (21.2)	2180 (20.0)	0.031
Steroids	160 (1.4)	235 (1.3)	0.010	148 (1.4)	156 (1.4)	0.007
LVEF measurement method						
Angiogram	4108 (35.8)	8970 (48.8)	0.266	4051 (37.2)	4461 (41.0)	0.075
Radionuclide	156 (1.4)	52 (0.3)	0.119	67 (0.6)	51 (0.5)	0.028
Echocardiogram	7076 (61.6)	9218 (50.1)	0.233	6646 (61.0)	6271 (57.6)	0.069
MRI	4 (0.0)	3 (0.0)	0.012	4 (0.0)	2 (0.0)	0.014
Elective	7677 (66.8)	12254 (68.2)	0.031	7292 (67.0)	7359 (67.6)	0.013
Urgent	3574 (31.1)	5506 (29.9)	0.026	3376 (31.0)	3313 (30.4)	0.013
Number of grafts, mean ± SD	3.1 ± 0.9	3.4 ± 1.0	0.368	3.1 ± 0.9	3.2 ± 0.9	0.094
On-pump surgery	10888 (94.7)	17113 (93.0)	0.072	10310 (94.7)	10265 (94.3)	0.016

SMD, standardized mean difference; PSM, propensity score matching; MAG, multiple arterial grafting; SAG, single arterial grafting; LVEF, left ventricular ejection fraction; CCS, Canadian Cardiovascular Society classification; New York Heart Association classification; MRI, magnetic resonance imaging.

© 2025 Ren J et al. *JAMA Network Open.*

eTable 15. Patient Demographics of MAG and SVG Cohorts Before and After Propensity Score Matching (LVEF 46-60%)

	Before PSM			After PSM		
**Characteristics Patients, No. (%)**	**SAG**	**MAG**	**SMD**	**SAG**	**MAG**	**SMD**
Total sample size	8001	10979	-	7311	7311	-
Age, mean ± SD	67.0 ± 10.2	65.0 ± 10.3	0.200	66.0 ± 10.7	66.6 ± 10.2	0.061
Male	6482 (81.0)	9110 (83.0)	0.051	5960 (81.5)	5986 (81.9)	0.010
Body mass index	29.2 ± 9.3	29.0 ± 5.8	0.026	29.2 ± 6.1	29.2 ± 10.2	0.009
Smoking history	5288 (66.1)	7222 (65.8)	0.007	4798 (65.6)	4822 (66.0)	0.007
Diabetes	3149 (39.4)	3785 (34.5)	0.101	2715 (37.1)	2754 (37.7)	0.011
Hypercholesterolemia	6538 (81.7)	8895 (81.0)	0.018	5937 (81.2)	5950 (81.4)	0.005
Creatinine, mean ± SD, μmol/L	107.3 ± 99.8	96.1 ± 63.8	0.134	96.2 ± 58.1	99.4 ± 74.7	0.026
Dialysis	222 (2.8)	88 (0.8)	0.149	83 (1.1)	103 (1.4)	0.031
Hypertension	6581 (82.3)	8566 (78.0)	0.106	5862 (80.2)	5862 (80.2)	0.029
Cerebrovascular event	863 (10.8)	991 (9.0)	0.059	708 (9.7)	732 (10.0)	0.012
Peripheral vascular disease	946 (11.8)	1126 (10.3)	0.050	820 (11.2)	830 (11.4)	0.005
Respiratory disease	1054 (13.2)	1221 (11.1)	0.063	912 (12.5)	913 (12.5)	0
Myocardial infarction	4770 (59.6)	6332 (57.7)	0.040	4294 (58.7)	4305 (58.9)	0.003
Congestive heart failure	860 (10.8)	928 (8.5)	0.078	668 (9.1)	720 (9.9)	0.026
CCS ≥3	3191 (39.9)	4592 (41.8)	0.040	2938 (40.2)	2919 (39.9)	0.006
NYHA ≥3	1323 (16.5)	1722 (15.7)	0.023	1168 (16.0)	1163 (15.9)	0.002
Cardiogenic shock	74 (0.9)	55 (0.5)	0.050	49 (0.7)	50 (0.7)	0.002
Resuscitation	46 (0.6)	36 (0.3)	0.037	29 (0.4)	33 (0.5)	0.010
Arrhythmia	767 (9.6)	865 (7.9)	0.061	622 (8.5)	645 (8.8)	0.012
Left main disease	2266 (28.3)	2656 (24.2)	0.094	1930 (26.4)	1981 (27.1)	0.016
Number of diseased territories, mean ± SD	2.7 ± 0.5	2.7 ± 0.5	0.007	2.7 ± 0.5	2.7 ± 0.5	0.002
Single-vessel disease	97 (1.2)	209 (1.9)	0.056	115 (1.6)	96 (1.3)	0.019
Double-vessel disease	1803 (22.5)	2407 (21.9)	0.015	1629 (22.3)	1666 (22.8)	0.012
Triple-vessel disease	6062 (75.8)	8325 (75.8)	0.001	5536 (75.7)	5515 (75.4)	0.007
Perioperative medications						
Inotropes	123 (1.5)	685 (6.2)	0.032	104 (1.4)	105 (1.4)	0.001
Nitroglycerin	430 (5.4)	128 (1.2)	0.037	405 (5.5)	394 (5.4)	0.006
Anticoagulants	1913 (23.9)	2458 (22.4)	0.036	1706 (23.3)	1706 (23.3)	0
Steroids	128 (1.6)	136 (1.2)	0.031	104 (1.4)	110 (1.5)	0.007
LVEF measurement method						
Angiogram	2488 (31.1)	4636 (42.2)	0.233	2599 (35.6)	2419 (33.1)	0.050
Radionuclide	81 (1.0)	85 (0.8)	0.025	68 (0.9)	70 (1.0)	0.003
Echocardiogram	5399 (67.5)	6174 (56.2)	0.233	4616 (63.1)	4797 (65.6)	0.050
MRI	11 (0.1)	2 (0.0)	0.043	2 (0.0)	3 (0.0)	0.010
Elective	4701 (58.8)	6690 (60.9)	0.045	4411 (60.3)	4371 (59.8)	0.011
Urgent	3037 (38.0)	4020 (36.6)	0.028	2704 (37.0)	2720 (37.2)	0.005
Number of grafts, mean ± SD	3.1 ± 0.9	3.4 ± 1.0	0.309	3.3 ± 0.9	3.2 ± 0.9	0.097
On-pump surgery	7638 (95.5)	9933 (90.5)	0.196	6908 (94.5)	6951 (95.1)	0.020

SMD, standardized mean difference; PSM, propensity score matching; MAG, multiple arterial grafting; SAG, single arterial grafting; LVEF, left ventricular ejection fraction; CCS, Canadian Cardiovascular Society classification; New York Heart Association classification; MRI, magnetic resonance imaging.

© 2025 Ren J et al. *JAMA Network Open.*

eTable 16. Patient Demographics of MAG and SVG Cohorts Before and After Propensity Score Matching (LVEF 30-45%)

	Before PSM			After PSM		
**Characteristics Patients, No. (%)**	**SAG**	**MAG**	**SMD**	**SAG**	**MAG**	**SMD**
Total sample size	3946	4694	-	3366	3366	-
Age, mean ± SD	66.6 ± 10.6	65.3 ± 10.6	0.123	66.2 ± 10.7	66.1 ± 10.5	0.012
Male	3211 (81.4)	3974 (84.7)	0.088	2795 (83.0)	2799 (83.2)	0.003
Body mass index	29.0 ± 9.6	28.9 ± 5.7	0.012	29.0 ± 10.1	29.0 ± 6.1	0.002
Smoking history	2719 (68.9)	3305 (70.4)	0.033	2357 (70.0)	2332 (69.3)	0.016
Diabetes	1803 (45.7)	1915 (40.8)	0.099	1462 (43.4)	1444 (42.9)	0.011
Hypercholesterolemia	3145 (79.7)	3662 (78.0)	0.041	2660 (79.8)	2635 (78.3)	0.018
Creatinine, mean ± SD, μmol/L	118.7 ± 121.3	102.4 ± 73.8	0.162	97.6 ± 56.7	95.3 ± 46.3	0.031
Dialysis	152 (3.9)	52 (1.1)	0.177	64 (1.9)	51 (1.5)	0.037
Hypertension	6444 (82.2)	3652 (77.8)	0.109	2715 (81.0)	2709 (80.1)	0.004
Cerebrovascular event	516 (13.1)	547 (11.7)	0.043	420 (12.5)	422 (12.5)	0.005
Peripheral vascular disease	586 (14.9)	675 (14.4)	0.013	486 (14.4)	505 (15.0)	0.016
Respiratory disease	644 (16.3)	689 (14.7)	0.045	532 (15.8)	509 (15.1)	0.019
Myocardial infarction	3039 (77.0)	3531 (75.2)	0.042	2568 (76.3)	2539 (75.4)	0.020
Congestive heart failure	1170 (29.7)	1128 (24.0)	0.127	908 (27.0)	886 (26.3)	0.015
CCS ≥3	1690 (42.8)	2104 (44.8)	0.040	1432 (42.5)	1441 (42.8)	0.005
NYHA ≥3	1072 (27.2)	1190 (25.4)	0.041	871 (25.9)	882 (26.2)	0.008
Cardiogenic shock	156 (3.9)	100 (2.1)	0.106	100 (3.0)	87 (2.6)	0.027
Resuscitation	71 (1.8)	47 (1.0)	0.068	52 (1.5)	41 (1.2)	0.033
Arrhythmia	586 (14.9)	602 (12.8)	0.059	482 (14.3)	451 (13.4)	0.028
Left main disease	1125 (28.5)	1184 (25.2)	0.074	925 (27.5)	902 (26.9)	0.016
Number of diseased territories, mean ± SD	2.8 ± 0.5	2.8 ± 0.5	0.014	2.7 ± 0.5	2.7 ± 0.5	0.013
Single-vessel disease	34 (0.9)	66 (1.4)	0.051	32 (1.0)	34 (1.0)	0.005
Double-vessel disease	692 (17.5)	805 (17.2)	0.010	581 (17.3)	592 (17.6)	0.009
Triple-vessel disease	3204 (81.2)	3805 (81.1)	0.004	2740 (81.4)	2725 (81.0)	0.011
Perioperative medications						
Inotropes	156 (4.0)	86 (1.8)	0.127	97 (2.9)	79 (2.4)	0.040
Nitroglycerin	293 (7.4)	389 (8.3)	0.032	250 (7.4)	258 (7.7)	0.009
Anticoagulants	1205 (30.5)	1215 (25.9)	0.104	973 (28.9)	942 (28.0)	0.021
Steroids	55 (1.4)	72 (1.5)	0.012	43 (1.3)	45 (1.3)	0.005
LVEF measurement method						
Angiogram	1096 (27.8)	1848 (39.4)	0.247	1004 (29.8)	1103 (32.8)	0.060
Radionuclide	85 (2.2)	80 (1.7)	0.033	69 (2.1)	66 (2.0)	0.007
Echocardiogram	2725 (69.1)	2729 (58.1)	0.228	2264 (67.3)	2171 (64.5)	0.056
MRI	24 (0.6)	15 (0.3)	0.043	15 (0.5)	13 (0.4)	0.010
Elective	2037 (51.6)	2641 (56.3)	0.093	1817 (54.0)	1835 (54.5)	0.011
Urgent	1659 (42.0)	1817 (38.7)	0.068	1361 (40.4)	1345 (40.0)	0.010
Number of grafts, mean ± SD	3.2 ± 0.9	3.5 ± 1.0	0.308	3.3 ± 0.9	3.4 ± 0.9	0.057
On-pump surgery	3790 (96.1)	4328 (92.2)	0.164	3214 (95.5)	3181 (94.5)	0.037

SMD, standardized mean difference; PSM, propensity score matching; MAG, multiple arterial grafting; SAG, single arterial grafting; LVEF, left ventricular ejection fraction; CCS, Canadian Cardiovascular Society classification; New York Heart Association classification; MRI, magnetic resonance imaging.

© 2025 Ren J et al. *JAMA Network Open.*

eTable 17. Patient Demographics of MAG and SVG Cohorts Before and After Propensity Score Matching (LVEF <30%)

	Before PSM			After PSM		
**Characteristics Patients, No. (%)**	**SAG**	**MAG**	**SMD**	**SAG**	**MAG**	**SMD**
Total sample size	1125	1005	-	765	765	-
Age, mean ± SD	65.4 ± 10.8	64.7 ± 10.6	0.065	65.3 ± 10.9	65.1 ± 10.5	0.019
Male	957 (85.1)	856 (85.2)	0.003	655 (85.6)	653 (85.4)	0.007
Body mass index	28.5 ± 13.1	28.4 ± 7.7	0.006	28.7 ± 15.3	28.7 ± 8.4	0.010
Smoking history	791 (70.3)	744 (74.0)	0.083	556 (72.7)	558 (72.9)	0.006
Diabetes	562 (50.0)	488 (48.6)	0.028	380 (49.7)	372 (48.6)	0.021
Hypercholesterolemia	873 (77.6)	777 (77.3)	0.007	587 (76.7)	585 (76.5)	0.006
Creatinine, mean ± SD, µmol/L	121.0 ± 107.6	108.0 ± 62.9	0.147	108.9 ± 46.7	67.3 ± 31.9	0.016
Dialysis	44 (3.9)	12 (1.2)	0.173	11 (1.4)	12 (1.6)	0.012
Hypertension	870 (77.3)	755 (75.1)	0.052	581 (75.9)	581 (75.9)	0
Cerebrovascular event	142 (12.6)	107 (10.7)	0.062	85 (11.1)	86 (11.2)	0.004
Peripheral vascular disease	175 (15.6)	140 (13.9)	0.046	112 (14.6)	116 (15.2)	0.015
Respiratory disease	180 (16.0)	156 (15.2)	0.013	112 (14.6)	116 (15.2)	0.014
Myocardial infarction	872 (77.5)	796 (79.2)	0.041	593 (77.5)	599 (78.3)	0.019
Congestive heart failure	583 (51.8)	523 (52.0)	0.004	398 (52.0)	391 (51.1)	0.018
CCS ≥3	481 (42.8)	489 (48.7)	0.119	345 (45.1)	364 (47.6)	0.050
NYHA ≥3	534 (47.5)	467 (46.5)	0.020	359 (46.9)	357 (46.7)	0.005
Cardiogenic shock	128 (11.4)	63 (6.3)	0.181	62 (8.1)	60 (7.8)	0.011
Resuscitation	52 (4.6)	33 (3.3)	0.069	32 (4.2)	28 (3.7)	0.029
Arrhythmia	211 (18.8)	204 (20.3)	0.039	146 (19.1)	146 (19.0)	0
Left main disease	346 (30.7)	293 (29.2)	0.035	250 (32.7)	238 (31.1)	0.017
Number of diseased territories, mean ± SD	2.8 ± 0.5	2.8 ± 0.4	0.098	2.5 ± 0.4	2.4 ± 0.4	0.019
Single-vessel disease	7 (0.6)	10 (1.0)	0.042	6 (0.8)	8 (1.1)	0.026
Double-vessel disease	193 (17.2)	137 (13.6)	0.098	120 (15.7)	113 (14.8)	0.027
Triple-vessel disease	917 (81.5)	856 (85.2)	0.098	636 (83.1)	642 (83.9)	0.022
Perioperative medications						
Inotropes	144 (12.8)	73 (7.3)	0.185	70 (9.2)	66 (8.6)	0.020
Nitroglycerin	114 (10.1)	108 (10.8)	0.020	74 (9.7)	80 (10.5)	0.025
Anticoagulants	377 (33.5)	353 (35.1)	0.034	250 (32.7)	266 (34.8)	0.044
Steroids	20 (1.8)	23 (2.3)	0.036	17 (2.2)	15 (2.0)	0.018
LVEF measurement method						
Angiogram	232 (20.6)	376 (37.4)	0.377	210 (27.5)	217 (28.4)	0.019
Radionuclide	66 (5.9)	45 (4.5)	0.063	49 (6.4)	43 (5.6)	0.038
Echocardiogram	808 (71.8)	563 (56.0)	0.334	493 (64.4)	489 (63.9)	0.011
MRI	16 (1.4)	11 (1.1)	0.029	10 (1.3)	10 (1.3)	0
Elective	529 (47.0)	492 (48.9)	0.039	383 (50.1)	369 (48.2)	0.037
Urgent	458 (40.7)	417 (41.5)	0.016	303 (39.6)	315 (41.2)	0.032
Number of grafts, mean ± SD	3.3 ± 1.0	3.7 ± 1.0	0.345	3.5 ± 1.0	3.6 ± 1.0	0.069
On-pump surgery	1075 (95.6)	958 (95.3)	0.011	729 (95.3)	732 (95.7)	0.019

SMD, standardized mean difference; PSM, propensity score matching; MAG, multiple arterial grafting; SAG, single arterial grafting; LVEF, left ventricular ejection fraction; CCS, Canadian Cardiovascular Society classification; New York Heart Association classification; MRI, magnetic resonance imaging.

© 2025 Ren J et al. *JAMA Network Open.*

eFigure 1. Summary of the Analytical Steps

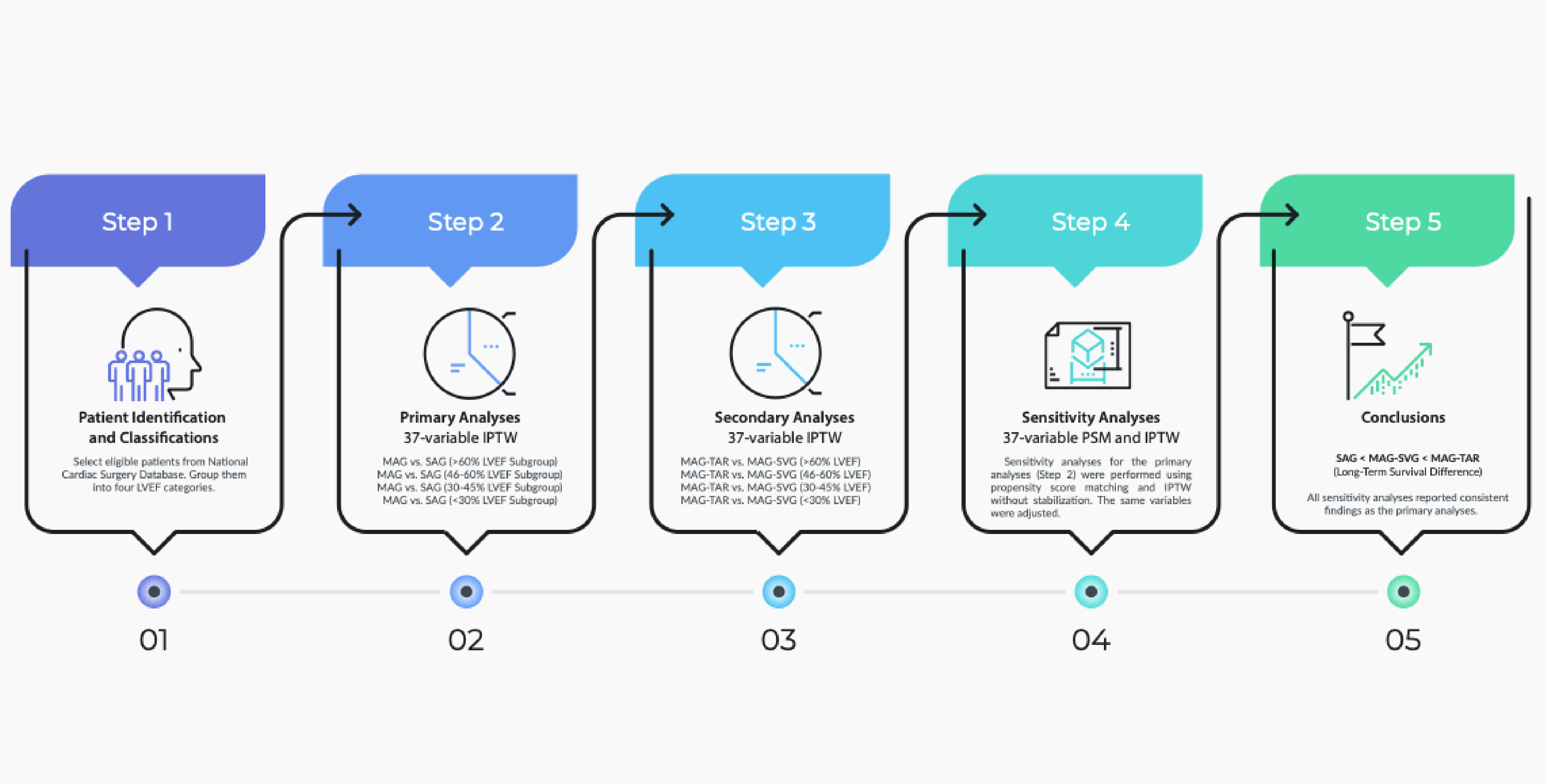

All analyses in this study were adjusted for 37 variables obtained from the national registry. MAG patients received more than one arterial conduit during surgery, while SAG patients received only one. Within the MAG group, MAG-TAR patients underwent multiple arterial grafting without the use of any supplementary saphenous vein grafts, whereas MAG-SVG patients received multiple arterial grafts supplemented by at least one saphenous vein graft. TAR, total arterial revascularization; MAG, multiple arterial grafting; SAG, single arterial grafting; LVEF, left ventricular ejection fraction; IPTW, inverse probability of treatment weighting; PSM, propensity score matching.

© 2025 Ren J et al. *JAMA Network Open.*

eFigure 2. Histogram of Propensity Score Distribution in MAG vs SAG Subgroup (LVEF >60%) Before and After Inverse Probability Weighting

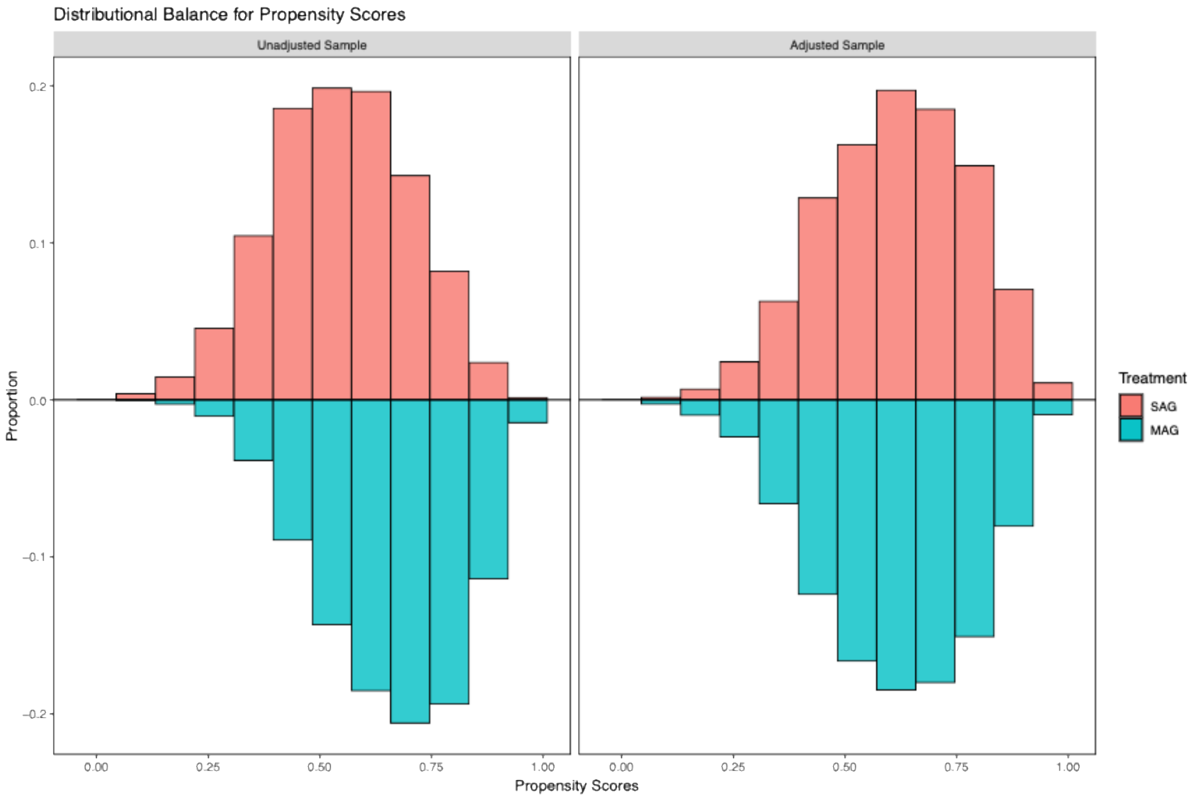

LVEF, left ventricular ejection fraction, MAG, multiple arterial grafting; SAG, single arterial grafting.

© 2025 Ren J et al. *JAMA Network Open.*

eFigure 3. Histogram of Propensity Score Distribution in MAG vs SAG Subgroup (LVEF 46-60%) Before and After Inverse Probability Weighting

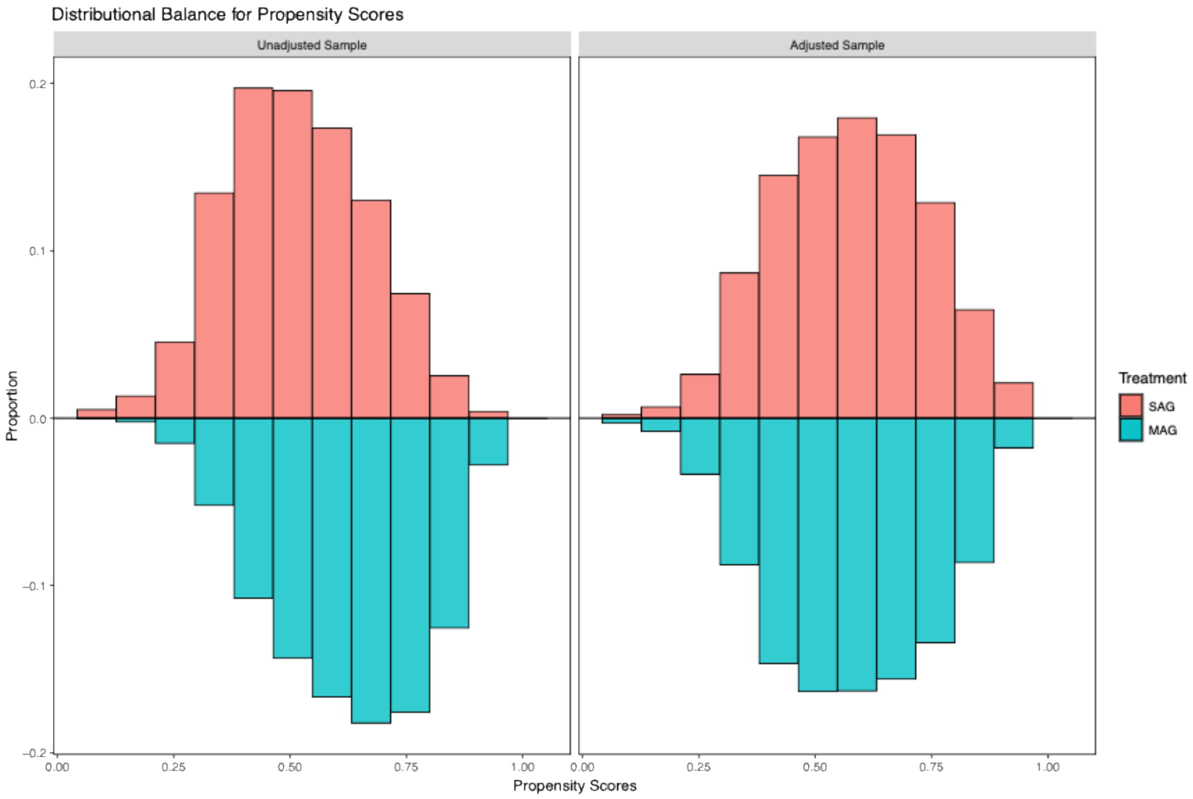

LVEF, left ventricular ejection fraction, MAG, multiple arterial grafting; SAG, single arterial grafting.

© 2025 Ren J et al. *JAMA Network Open.*

eFigure 4. Histogram of Propensity Score Distribution in MAG vs SAG Subgroup (LVEF 30-45%) Before and After Inverse Probability Weighting

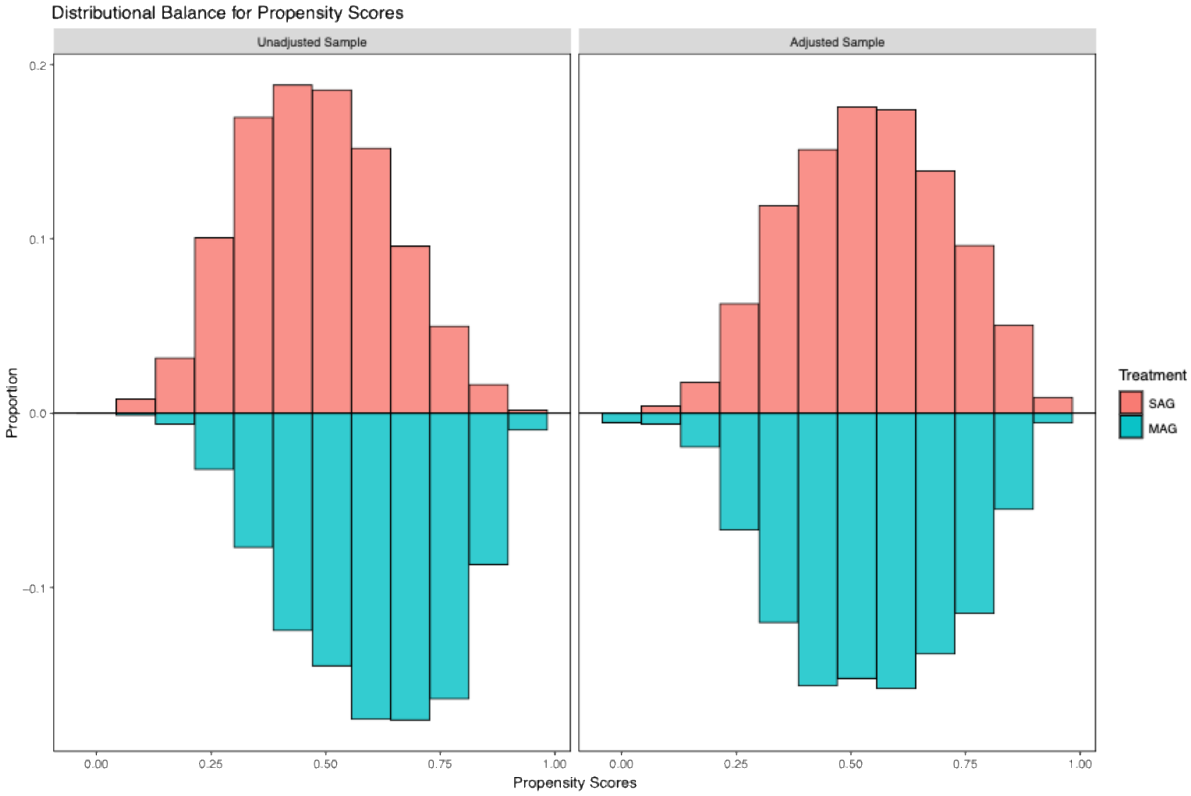

LVEF, left ventricular ejection fraction, MAG, multiple arterial grafting; SAG, single arterial grafting.

© 2025 Ren J et al. *JAMA Network Open.*

eFigure 5. Histogram of Propensity Score Distribution in MAG vs SAG Subgroup (LVEF <30%) Before and After Inverse Probability Weighting

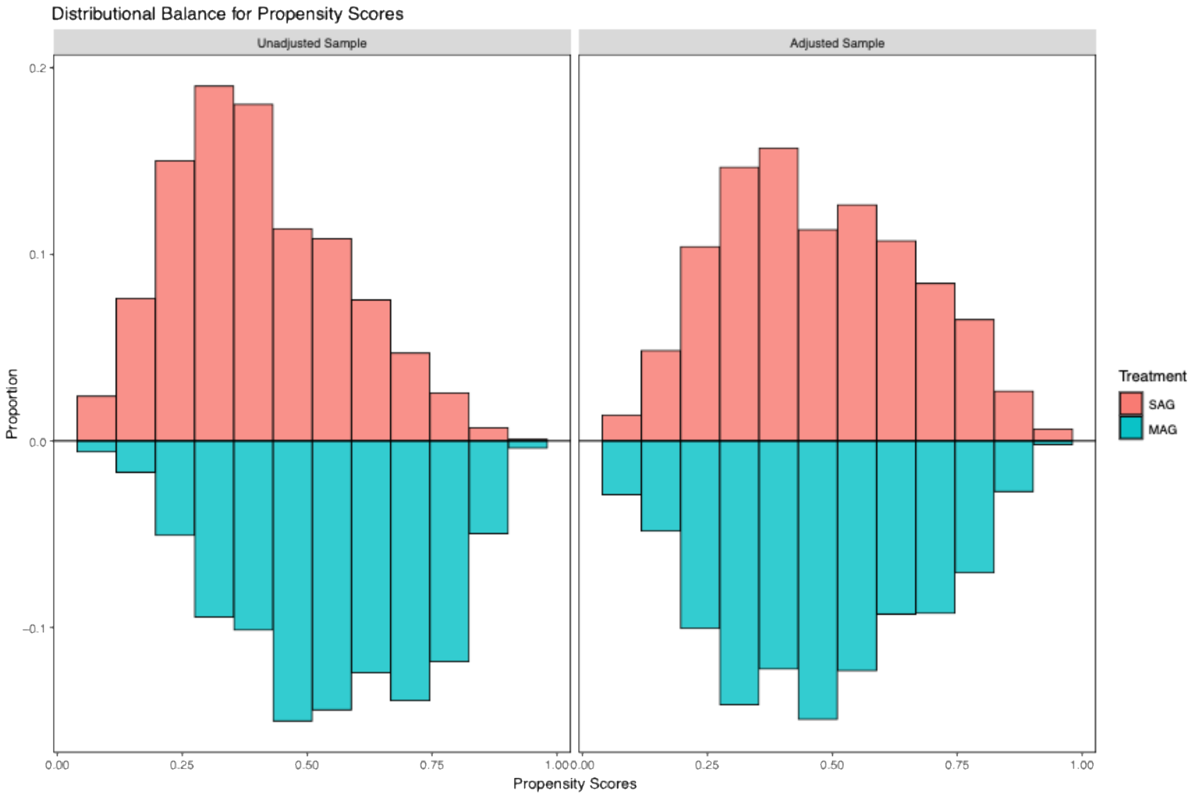

LVEF, left ventricular ejection fraction, MAG, multiple arterial grafting; SAG, single arterial grafting.

© 2025 Ren J et al. *JAMA Network Open.*

eFigure 6. Absolute Standardized Mean Differences of Covariates Before and After Adjustment in the MAG vs SAG Comparison (LVEF >60%)

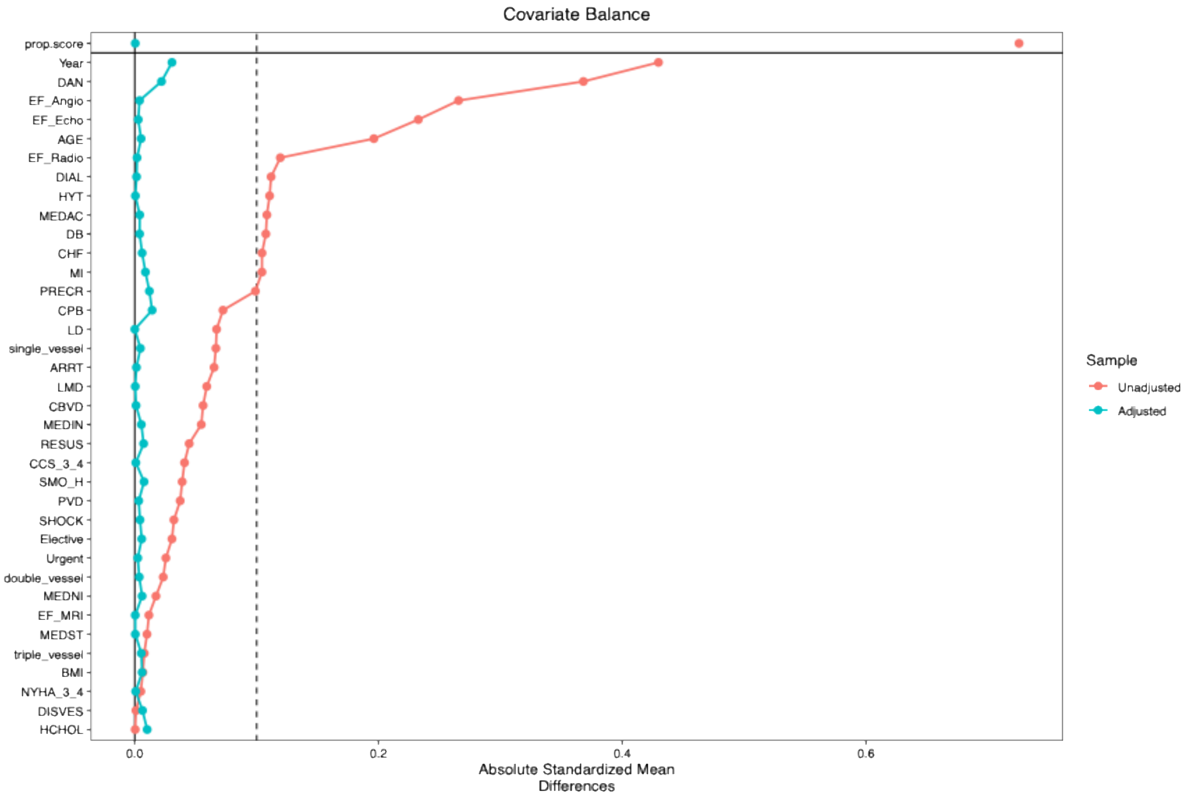

DAN, number of grafts; triple_vessel, triple vessel disease; DISVES, number of diseased territories; double_vessel, double vessel disease; single_vessel, single vessel disease, CPB, cardiopulmonary bypass; Year, year of operation; AGE, patient age; MEDIN, inotropes; MEDAC, anticoagulation therapy; DB, diabetes mellitus; MI, myocardial infarction; NYHA_3_4, New York Heart Association Classification ≥3; HYT, hypertension; MEDNI, intravenous nitrates; DIAL, dialysis; PRECR, preoperative creatinine level; EF_Echo, echocardiogram; CCS_3_4, Canadian Cardiovascular Society (CCS) classification ≥3; SMO_H, smoking history; EF_Angio, angiography; CBVD, cerebrovascular disease; RESUS, resuscitation; LMD, left main disease; MEDST, steroids; ARRT, arrhythmia; CHF, history of congestive heart failure; HCHOL, hypercholesterolaemia; LD, respiratory disease; EF_MRI, magnetic resonance imaging; PVD, peripheral vascular disease; EF_Radio, nuclear imaging; BMI, body mass index.

© 2025 Ren J et al. *JAMA Network Open.*

eFigure 7. Absolute Standardized Mean Differences of Covariates Before and After Adjustment in the MAG vs SAG Comparison (LVEF 46-60%)

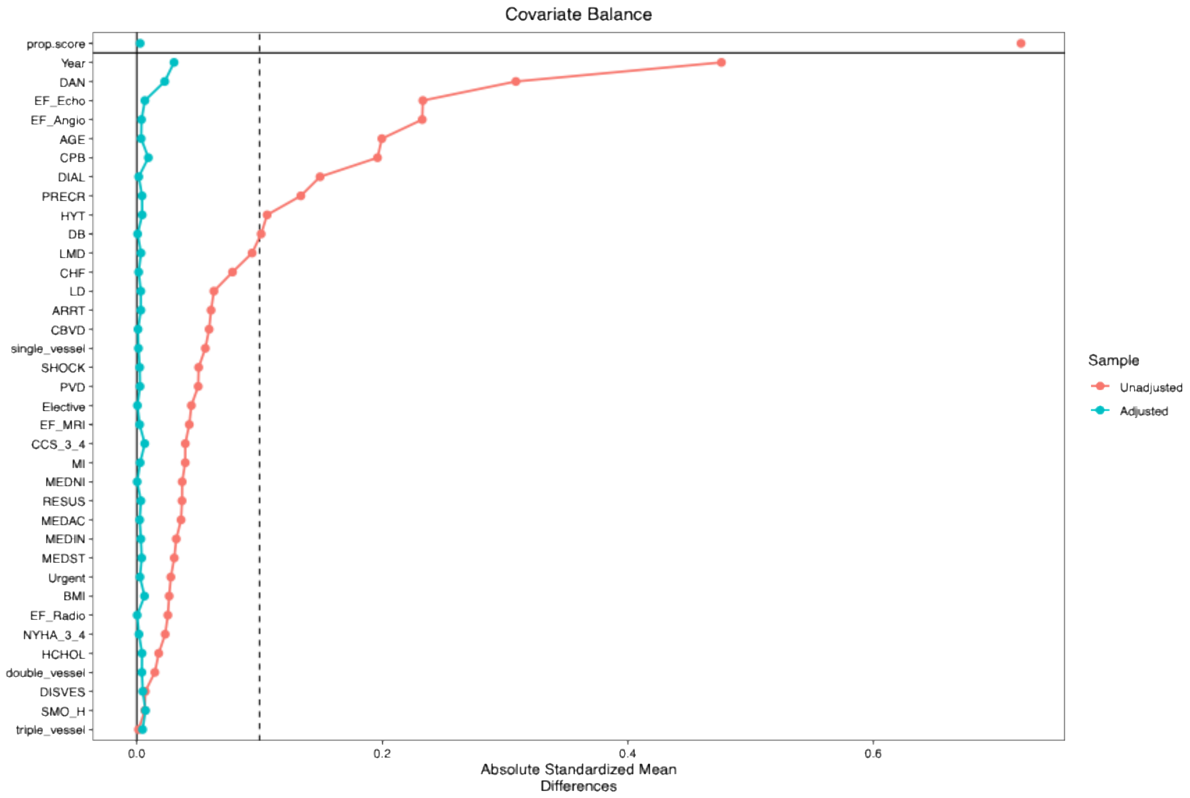

DAN, number of grafts; triple_vessel, triple vessel disease; DISVES, number of diseased territories; double_vessel, double vessel disease; single_vessel, single vessel disease, CPB, cardiopulmonary bypass; Year, year of operation; AGE, patient age; MEDIN, inotropes; MEDAC, anticoagulation therapy; DB, diabetes mellitus; MI, myocardial infarction; NYHA_3_4, New York Heart Association Classification ≥3; HYT, hypertension; MEDNI, intravenous nitrates; DIAL, dialysis; PRECR, preoperative creatinine level; EF_Echo, echocardiogram; CCS_3_4, Canadian Cardiovascular Society (CCS) classification ≥3; SMO_H, smoking history; EF_Angio, angiography; CBVD, cerebrovascular disease; RESUS, resuscitation; LMD, left main disease; MEDST, steroids; ARRT, arrhythmia; CHF, history of congestive heart failure; HCHOL, hypercholesterolaemia; LD, respiratory disease; EF_MRI, magnetic resonance imaging; PVD, peripheral vascular disease; EF_Radio, nuclear imaging; BMI, body mass index.

© 2025 Ren J et al. *JAMA Network Open.*

eFigure 8. Absolute Standardized Mean Differences of Covariates Before and After Adjustment in the MAG vs SAG Comparison (LVEF 30-45%)

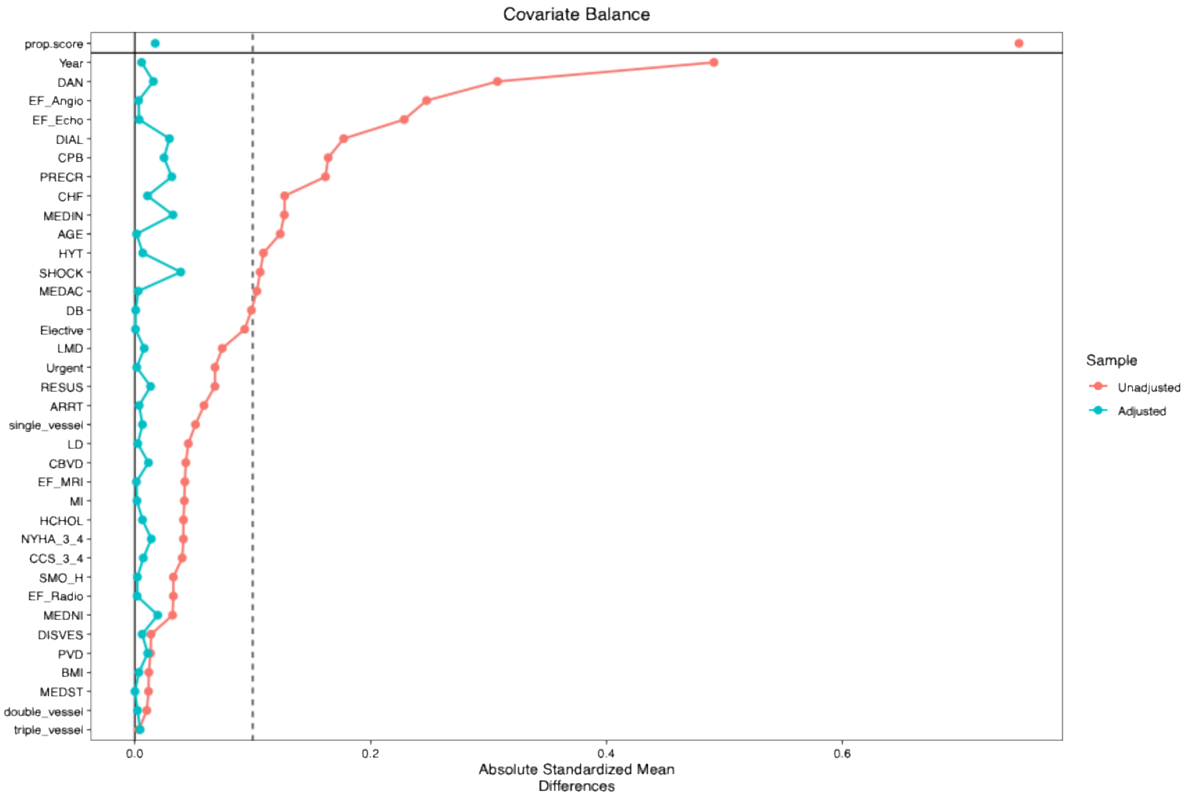

DAN, number of grafts; triple_vessel, triple vessel disease; DISVES, number of diseased territories; double_vessel, double vessel disease; single_vessel, single vessel disease, CPB, cardiopulmonary bypass; Year, year of operation; AGE, patient age; MEDIN, inotropes; MEDAC, anticoagulation therapy; DB, diabetes mellitus; MI, myocardial infarction; NYHA_3_4, New York Heart Association Classification ≥3; HYT, hypertension; MEDNI, intravenous nitrates; DIAL, dialysis; PRECR, preoperative creatinine level; EF_Echo, echocardiogram; CCS_3_4, Canadian Cardiovascular Society (CCS) classification ≥3; SMO_H, smoking history; EF_Angio, angiography; CBVD, cerebrovascular disease; RESUS, resuscitation; LMD, left main disease; MEDST, steroids; ARRT, arrhythmia; CHF, history of congestive heart failure; HCHOL, hypercholesterolaemia; LD, respiratory disease; EF_MRI, magnetic resonance imaging; PVD, peripheral vascular disease; EF_Radio, nuclear imaging; BMI, body mass index.

© 2025 Ren J et al. *JAMA Network Open.*

eFigure 9. Absolute Standardized Mean Differences of Covariates Before and After Adjustment in the MAG vs SAG Comparison (LVEF <30%)

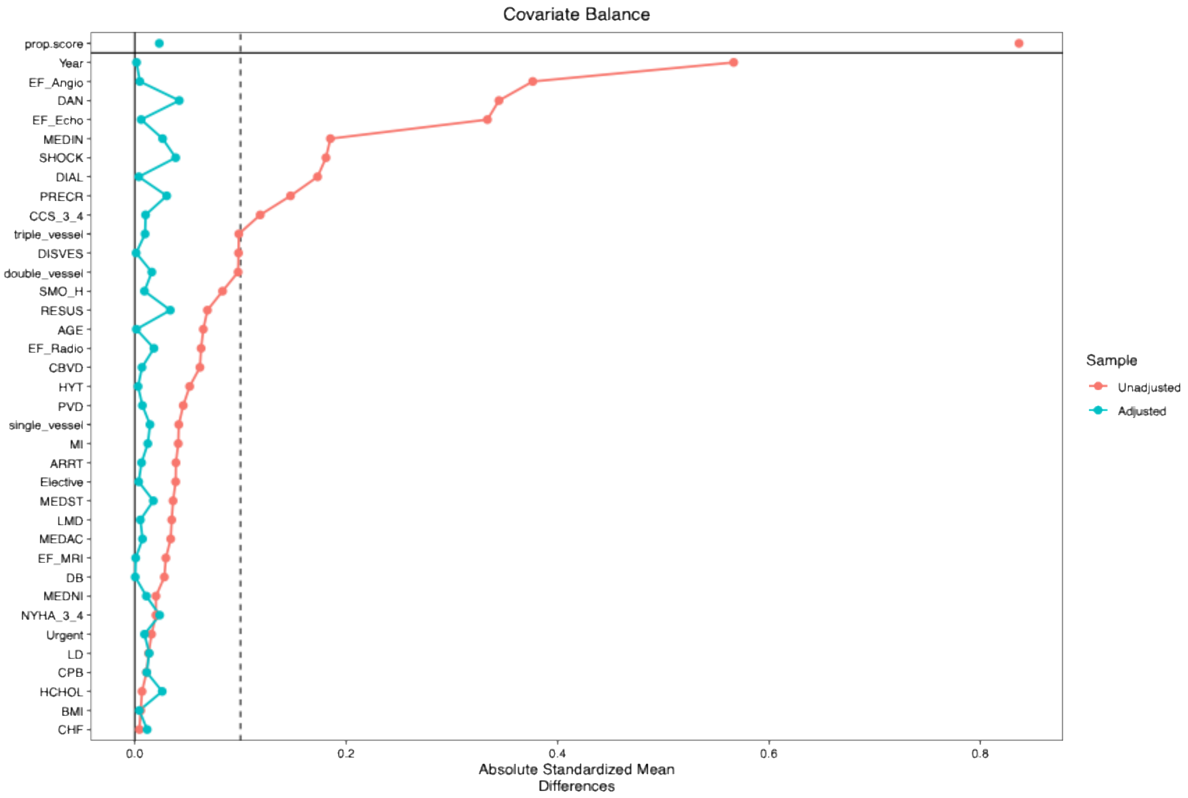

DAN, number of grafts; triple_vessel, triple vessel disease; DISVES, number of diseased territories; double_vessel, double vessel disease; single_vessel, single vessel disease, CPB, cardiopulmonary bypass; Year, year of operation; AGE, patient age; MEDIN, inotropes; MEDAC, anticoagulation therapy; DB, diabetes mellitus; MI, myocardial infarction; NYHA_3_4, New York Heart Association Classification ≥3; HYT, hypertension; MEDNI, intravenous nitrates; DIAL, dialysis; PRECR, preoperative creatinine level; EF_Echo, echocardiogram; CCS_3_4, Canadian Cardiovascular Society (CCS) classification ≥3; SMO_H, smoking history; EF_Angio, angiography; CBVD, cerebrovascular disease; RESUS, resuscitation; LMD, left main disease; MEDST, steroids; ARRT, arrhythmia; CHF, history of congestive heart failure; HCHOL, hypercholesterolaemia; LD, respiratory disease; EF_MRI, magnetic resonance imaging; PVD, peripheral vascular disease; EF_Radio, nuclear imaging; BMI, body mass index.

© 2025 Ren J et al. *JAMA Network Open.*

eFigure 10. Histogram of Propensity Score Distribution in MAG-TAR vs MAG-SVG Subgroup (LVEF >60%) Before and After Inverse Probability Weighting

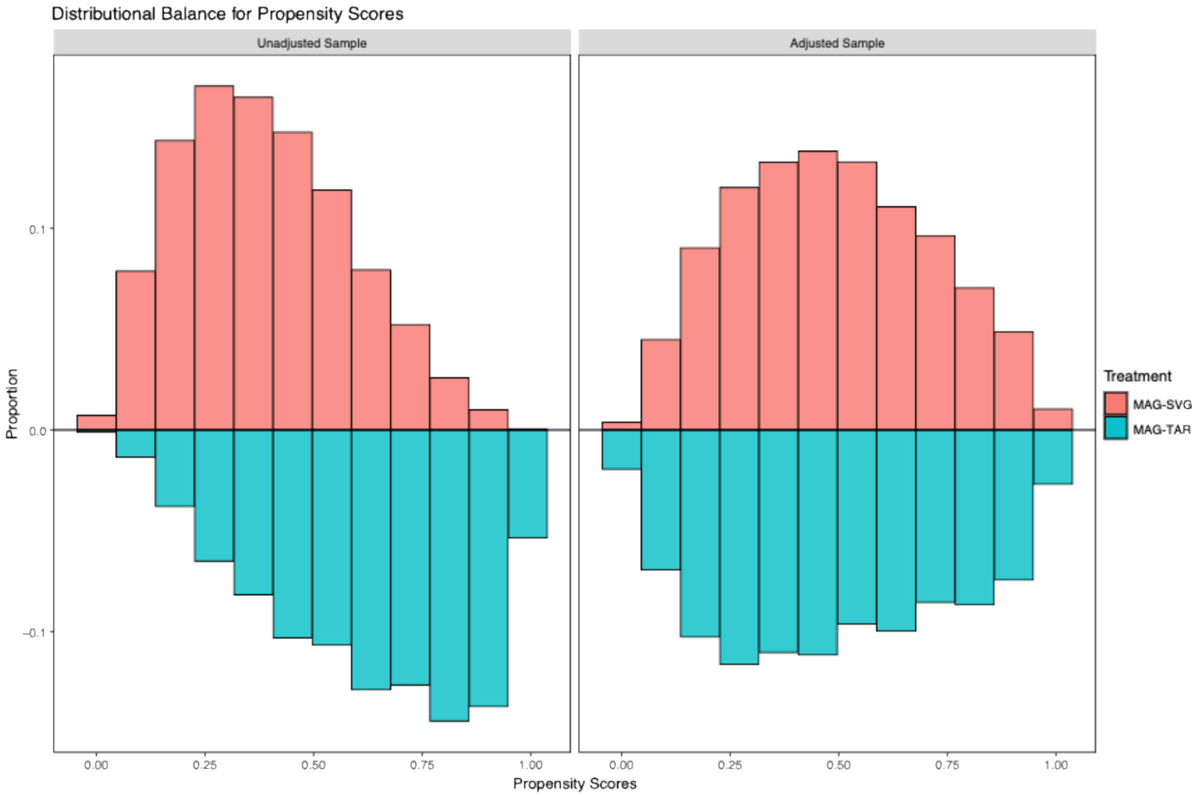

The MAG-TAR patient group received multiple arterial grafts without any supplementary saphenous vein grafts, whereas the MAG-SVG group received multiple arterial grafts with at least one saphenous vein graft. LVEF, left ventricular ejection fraction, MAG, multiple arterial grafting; TAR, total arterial revascularization; SVG, saphenous vein graft.

© 2025 Ren J et al. *JAMA Network Open.*

eFigure 11. Histogram of Propensity Score Distribution in MAG-TAR vs MAG-SVG Subgroup (LVEF 46-60%) Before and After Inverse Probability Weighting

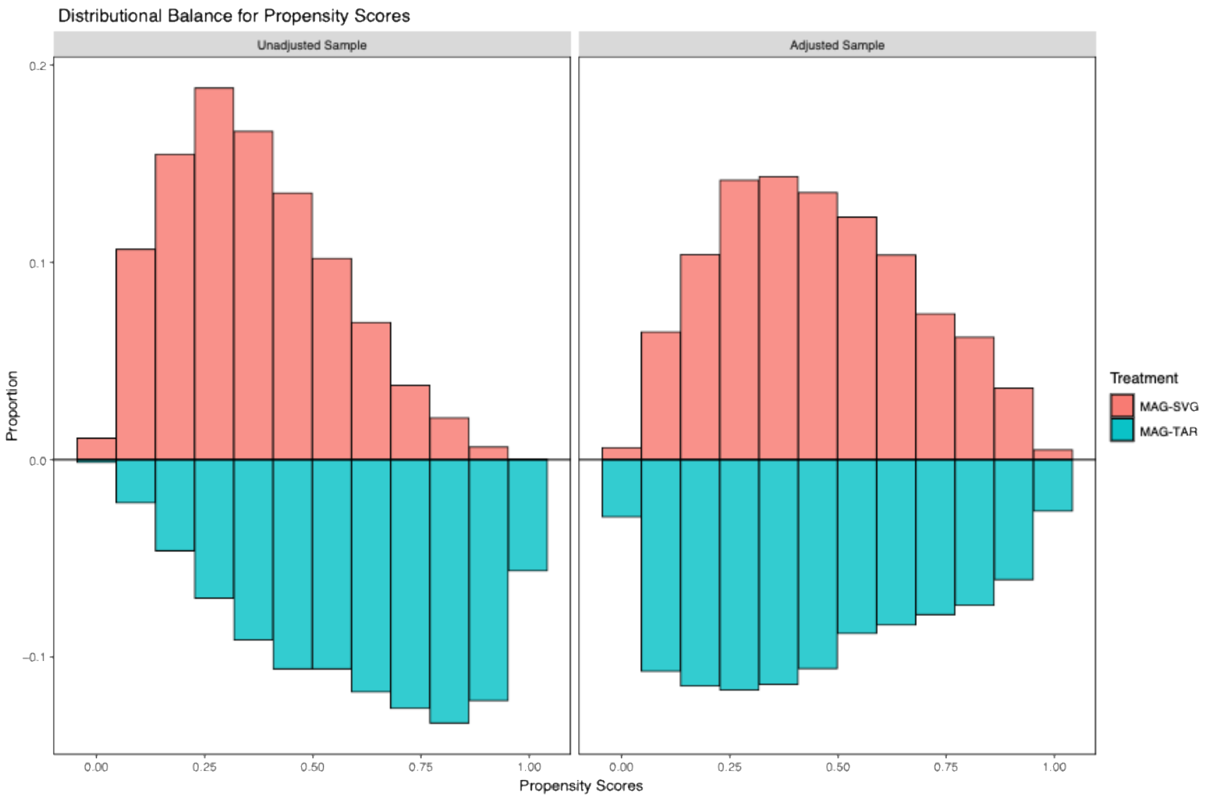

The MAG-TAR patient group received multiple arterial grafts without any supplementary saphenous vein grafts, whereas the MAG-SVG group received multiple arterial grafts with at least one saphenous vein graft. LVEF, left ventricular ejection fraction, MAG, multiple arterial grafting; TAR, total arterial revascularization; SVG, saphenous vein graft.

© 2025 Ren J et al. *JAMA Network Open.*

eFigure 12. Histogram of Propensity Score Distribution in MAG-TAR vs MAG-SVG Subgroup (LVEF 30-45%) Before and After Inverse Probability Weighting

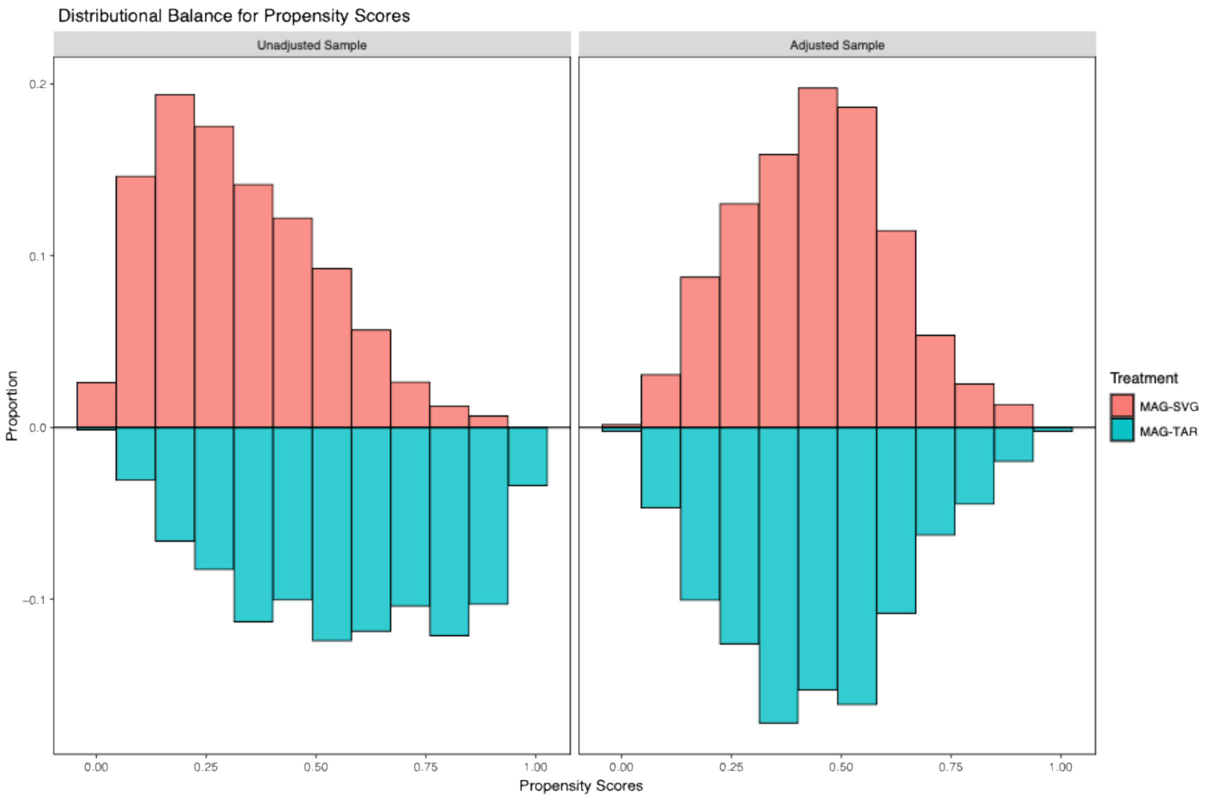

The MAG-TAR patient group received multiple arterial grafts without any supplementary saphenous vein grafts, whereas the MAG-SVG group received multiple arterial grafts with at least one saphenous vein graft. LVEF, left ventricular ejection fraction, MAG, multiple arterial grafting; TAR, total arterial revascularization; SVG, saphenous vein graft.

© 2025 Ren J et al. *JAMA Network Open.*

eFigure 13. Histogram of Propensity Score Distribution in MAG-TAR vs MAG-SVG Subgroup (LVEF <30%) Before and After Inverse Probability Weighting

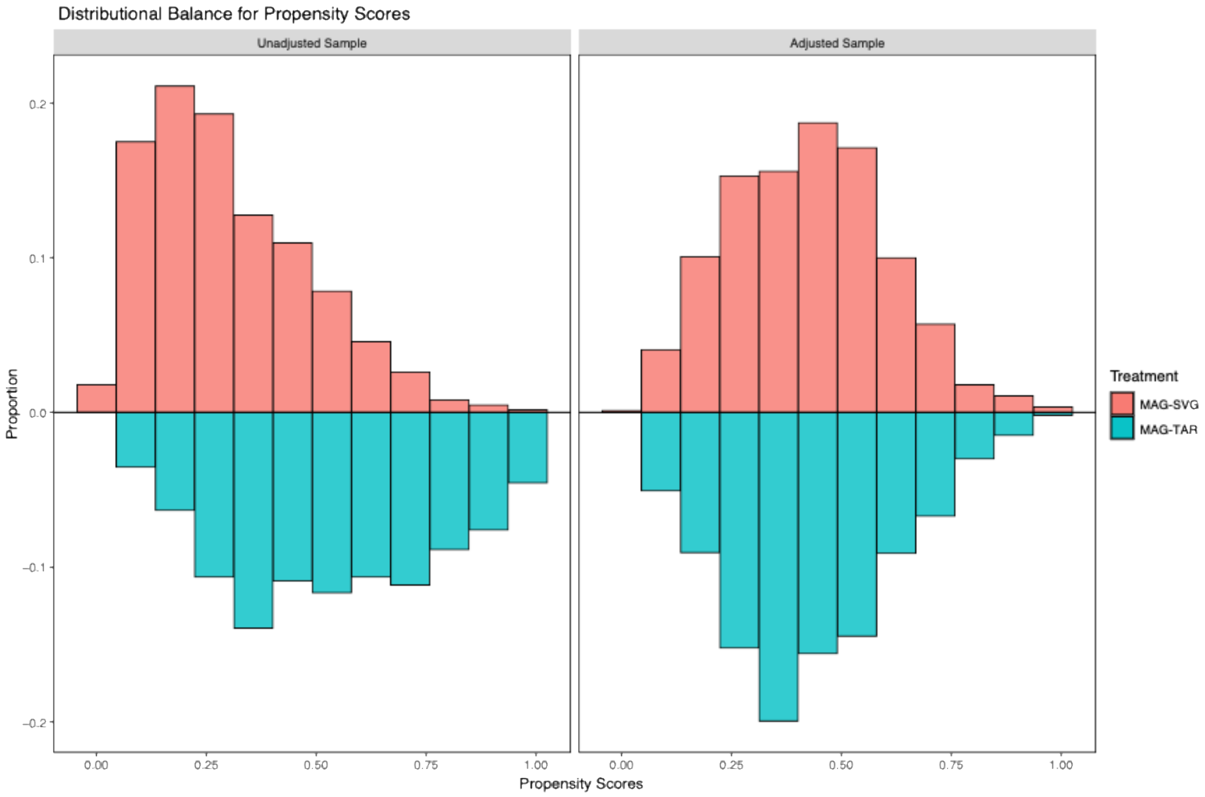

The MAG-TAR patient group received multiple arterial grafts without any supplementary saphenous vein grafts, whereas the MAG-SVG group received multiple arterial grafts with at least one saphenous vein graft. LVEF, left ventricular ejection fraction, MAG, multiple arterial grafting; TAR, total arterial revascularization; SVG, saphenous vein graft.

© 2025 Ren J et al. *JAMA Network Open.*

eFigure 14. Absolute Standardized Mean Differences of Covariates Before and After Adjustment in the MAG-TAR vs MAG-SVG Comparison (LVEF >60%)

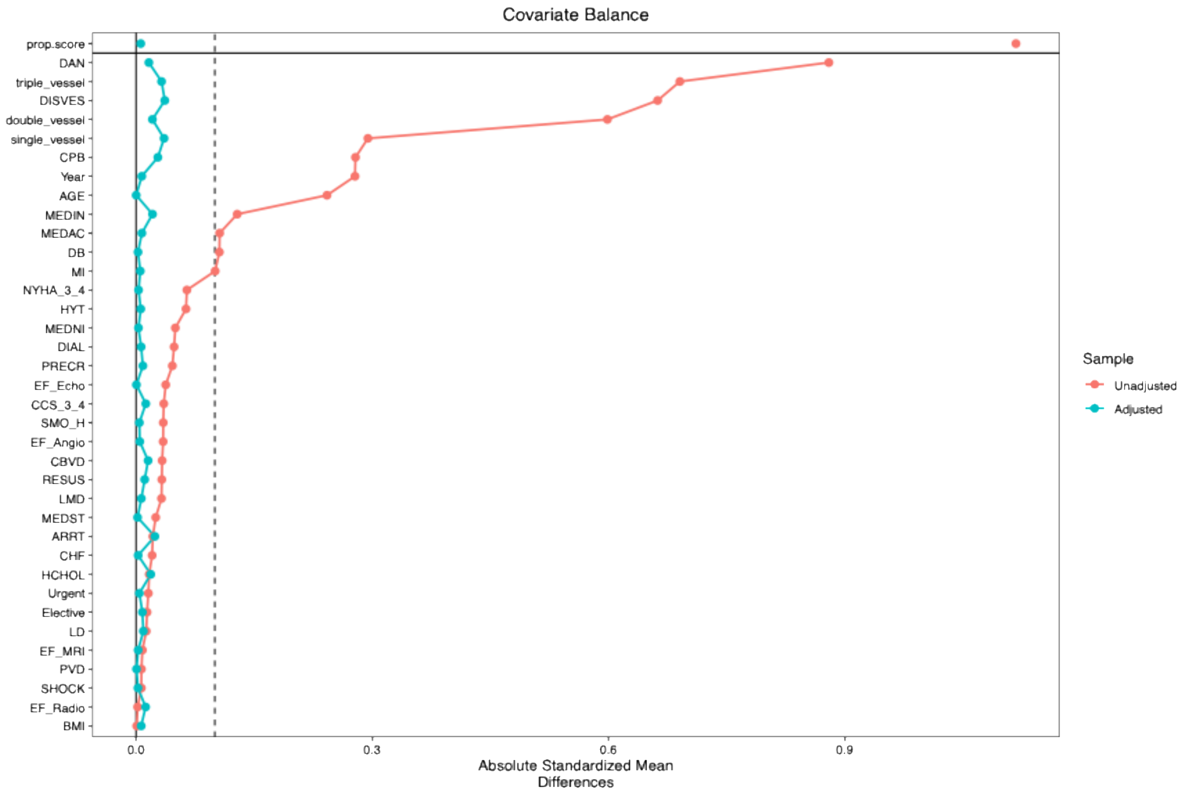

DAN, number of grafts; triple_vessel, triple vessel disease; DISVES, number of diseased territories; double_vessel, double vessel disease; single_vessel, single vessel disease, CPB, cardiopulmonary bypass; Year, year of operation; AGE, patient age; MEDIN, inotropes; MEDAC, anticoagulation therapy; DB, diabetes mellitus; MI, myocardial infarction; NYHA_3_4, New York Heart Association Classification ≥3; HYT, hypertension; MEDNI, intravenous nitrates; DIAL, dialysis; PRECR, preoperative creatinine level; EF_Echo, echocardiogram; CCS_3_4, Canadian Cardiovascular Society (CCS) classification ≥3; SMO_H, smoking history; EF_Angio, angiography; CBVD, cerebrovascular disease; RESUS, resuscitation; LMD, left main disease; MEDST, steroids; ARRT, arrhythmia; CHF, history of congestive heart failure; HCHOL, hypercholesterolaemia; LD, respiratory disease; EF_MRI, magnetic resonance imaging; PVD, peripheral vascular disease; EF_Radio, nuclear imaging; BMI, body mass index.

© 2025 Ren J et al. *JAMA Network Open.*

eFigure 15. Absolute Standardized Mean Differences of Covariates Before and After Adjustment in the MAG-TAR vs MAG-SVG Comparison (LVEF 46-60%)

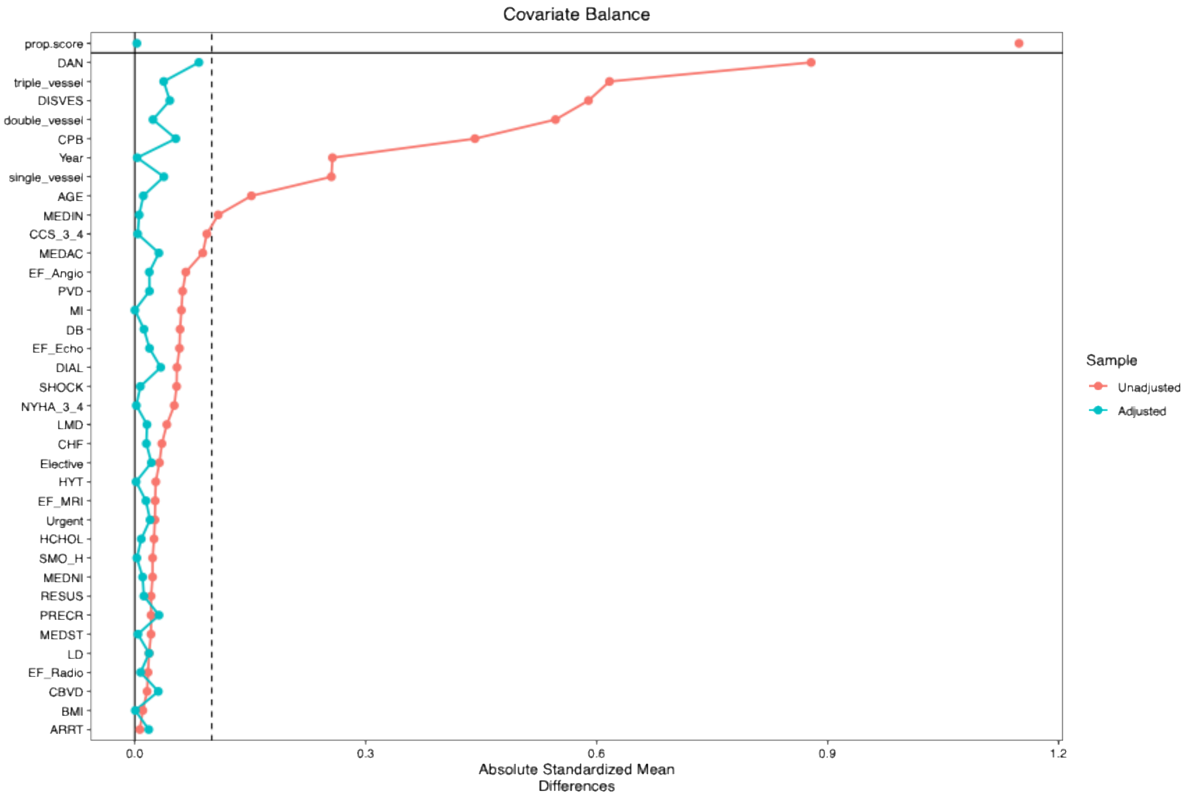

DAN, number of grafts; triple_vessel, triple vessel disease; DISVES, number of diseased territories; double_vessel, double vessel disease; single_vessel, single vessel disease, CPB, cardiopulmonary bypass; Year, year of operation; AGE, patient age; MEDIN, inotropes; MEDAC, anticoagulation therapy; DB, diabetes mellitus; MI, myocardial infarction; NYHA_3_4, New York Heart Association Classification ≥3; HYT, hypertension; MEDNI, intravenous nitrates; DIAL, dialysis; PRECR, preoperative creatinine level; EF_Echo, echocardiogram; CCS_3_4, Canadian Cardiovascular Society (CCS) classification ≥3; SMO_H, smoking history; EF_Angio, angiography; CBVD, cerebrovascular disease; RESUS, resuscitation; LMD, left main disease; MEDST, steroids; ARRT, arrhythmia; CHF, history of congestive heart failure; HCHOL, hypercholesterolaemia; LD, respiratory disease; EF_MRI, magnetic resonance imaging; PVD, peripheral vascular disease; EF_Radio, nuclear imaging; BMI, body mass index.

© 2025 Ren J et al. *JAMA Network Open.*

eFigure 16. Absolute Standardized Mean Differences of Covariates Before and After Adjustment in the MAG-TAR vs MAG-SVG Comparison (LVEF 30-45%)

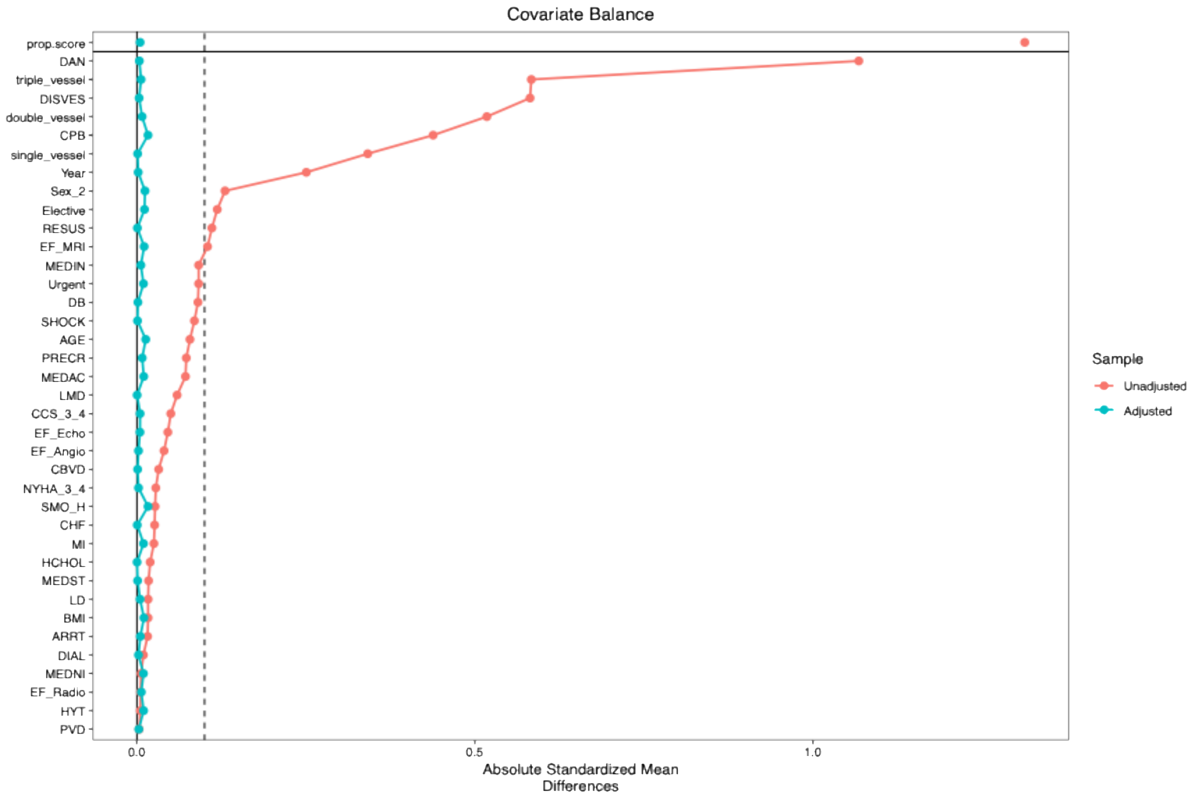

DAN, number of grafts; triple_vessel, triple vessel disease; DISVES, number of diseased territories; double_vessel, double vessel disease; single_vessel, single vessel disease, CPB, cardiopulmonary bypass; Year, year of operation; AGE, patient age; MEDIN, inotropes; MEDAC, anticoagulation therapy; DB, diabetes mellitus; MI, myocardial infarction; NYHA_3_4, New York Heart Association Classification ≥3; HYT, hypertension; MEDNI, intravenous nitrates; DIAL, dialysis; PRECR, preoperative creatinine level; EF_Echo, echocardiogram; CCS_3_4, Canadian Cardiovascular Society (CCS) classification ≥3; SMO_H, smoking history; EF_Angio, angiography; CBVD, cerebrovascular disease; RESUS, resuscitation; LMD, left main disease; MEDST, steroids; ARRT, arrhythmia; CHF, history of congestive heart failure; HCHOL, hypercholesterolaemia; LD, respiratory disease; EF_MRI, magnetic resonance imaging; PVD, peripheral vascular disease; EF_Radio, nuclear imaging; BMI, body mass index.

© 2025 Ren J et al. *JAMA Network Open.*

eFigure 17. Absolute Standardized Mean Differences of Covariates Before and After Adjustment in the MAG-TAR vs MAG-SVG Comparison (LVEF <30%)

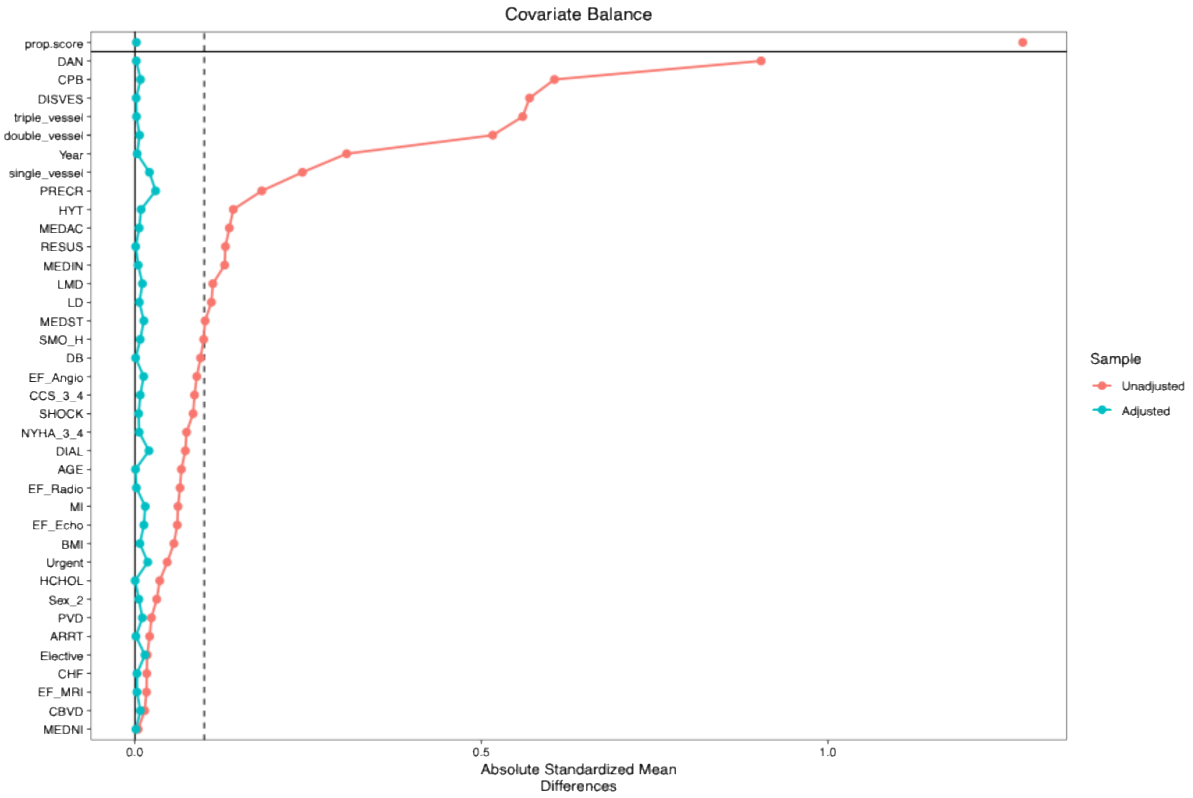

DAN, number of grafts; triple_vessel, triple vessel disease; DISVES, number of diseased territories; double_vessel, double vessel disease; single_vessel, single vessel disease, CPB, cardiopulmonary bypass; Year, year of operation; AGE, patient age; MEDIN, inotropes; MEDAC, anticoagulation therapy; DB, diabetes mellitus; MI, myocardial infarction; NYHA_3_4, New York Heart Association Classification ≥3; HYT, hypertension; MEDNI, intravenous nitrates; DIAL, dialysis; PRECR, preoperative creatinine level; EF_Echo, echocardiogram; CCS_3_4, Canadian Cardiovascular Society (CCS) classification ≥3; SMO_H, smoking history; EF_Angio, angiography; CBVD, cerebrovascular disease; RESUS, resuscitation; LMD, left main disease; MEDST, steroids; ARRT, arrhythmia; CHF, history of congestive heart failure; HCHOL, hypercholesterolaemia; LD, respiratory disease; EF_MRI, magnetic resonance imaging; PVD, peripheral vascular disease; EF_Radio, nuclear imaging; BMI, body mass index.

© 2025 Ren J et al. *JAMA Network Open.*
