# Supplementary material for: Survival Outcomes After Multiple vs Single Arterial Grafting Among Patients With Reduced Ejection Fraction
Source: JAMA Netw Open. 2025 Apr 10;8(4):e254508. doi: 10.1001/jamanetworkopen.2025.4508 (PMC11986767; doi:10.1001/jamanetworkopen.2025.4508)
Supplement: Supplement 2. — Data Sharing Statement [file jamanetwopen-e254508-s002.pdf]

Data Sharing Statement

Ren. Survival Outcomes After Multiple vs Single Arterial Grafting Among Patients With Reduced Ejection Fraction. *JAMA Netw Open*. Published April 10, 2025. doi:10.1001/jamanetworkopen.2025.4508

Data
Data available: No

Additional Information
Explanation for why data not available: IPD subject to national registry privacy regulation
